# A Comprehensive Methodology for Monitoring Evaporitic Mineral Precipitation and Hydrochemical Evolution of Saline Lakes: The Case of Lake Magadi Soda Brine (East African Rift Valley, Kenya)

**DOI:** 10.1021/acs.cgd.1c01391

**Published:** 2022-03-03

**Authors:** Melese Getenet, Juan Manuel García-Ruiz, Fermín Otálora, Franziska Emmerling, Dominik Al-Sabbagh, Cristóbal Verdugo-Escamilla

**Affiliations:** † Laboratorio de Estudios Cristalográficos, Instituto Andaluz de Ciencias de la Tierra (CSIC-UGR), Avenida de las Palmeras 4, Armilla, E-18100 Granada, Spain; ‡ Federal Institute for Materials Research and Testing (BAM), Richard-Willstätter-Straße 11, 12489 Berlin, Germany

## Abstract

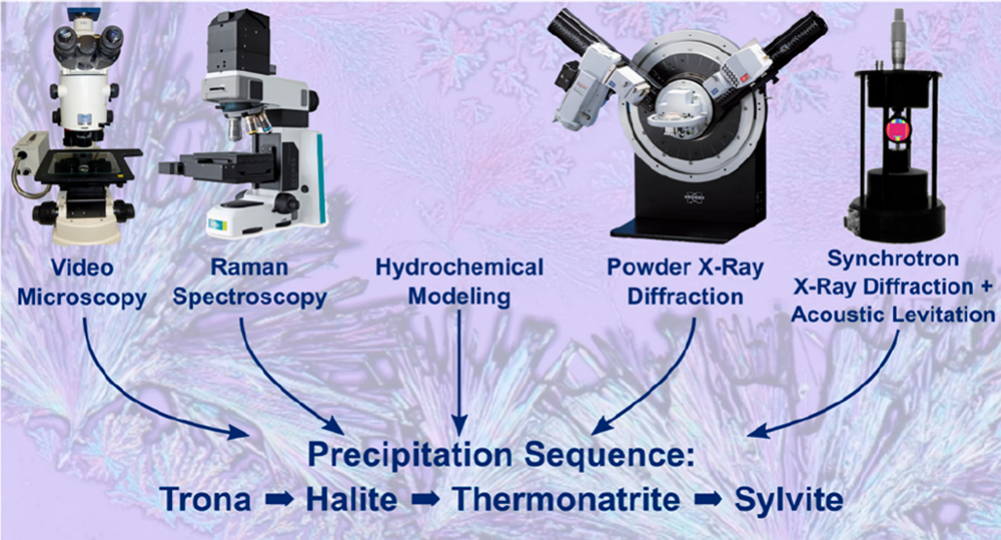

Lake Magadi, East African Rift Valley, is a hyperalkaline and saline soda lake highly enriched in Na^+^, K^+^, CO_3_^2–^, Cl^–^, HCO_3_^–^, and SiO_2_ and depleted in Ca^2+^ and Mg^2+^, where thick evaporite deposits and siliceous sediments have been forming for 100 000 years. The hydrogeochemistry and the evaporite deposits of soda lakes are subjects of growing interest in paleoclimatology, astrobiology, and planetary sciences. In Lake Magadi, different hydrates of sodium carbonate/bicarbonate and other saline minerals precipitate. The precipitation sequence of these minerals is a key for understanding the hydrochemical evolution, the paleoenvironmental conditions of ancient evaporite deposits, and industrial crystallization. However, accurate determination of the precipitation sequence of these minerals was challenging due to the dependency of the different hydrates on temperature, water activity, pH and pCO_2_, which could induce phase transformation and secondary mineral precipitation during sample handling. Here, we report a comprehensive methodology applied for monitoring the evaporitic mineral precipitation and hydrochemical evolution of Lake Magadi. Evaporation and mineral precipitations were monitored by using in situ video microscopy and synchrotron X-ray diffraction of acoustically levitated droplets. The mineral patterns were characterized by ex situ Raman spectroscopy, X-ray diffraction, and scanning electron microscopy. Experiments were coupled with thermodynamic models to understand the evaporation and precipitation-driven hydrochemical evolution of brines. Our results closely reproduced the mineral assemblages, patterns, and textural relations observed in the natural setting. Alkaline earth carbonates and fluorite were predicted to precipitate first followed by siliceous sediments. Among the salts, dendritic and acicular trona precipitate first via fractional crystallization—reminiscent of grasslike trona layers of Lake Magadi. Halite/villiaumite, thermonatrite, and sylvite precipitate sequentially after trona from residual brines depleted in HCO_3_^–^. The precipitation of these minerals between trona crystals resembles the precipitation process observed in the interstitial brines of the trona layers. Thermonatrite precipitation began after trona equilibrated with the residual brines due to the absence of excess CO_2_ input. We have shown that evaporation and mineral precipitation are the major drivers for the formation of hyperalkaline, saline, and SiO_2_-rich brines. The discrepancy between predicted and actual sulfate and phosphate ion concentrations implies the biological cycling of these ions. The combination of different in situ and ex situ methods and modeling is key to understanding the mineral phases, precipitation sequences, and textural relations of modern and ancient evaporite deposits. The synergy of these methods could be applicable in industrial crystallization and natural brines to reconstruct the hydrogeochemical and hydroclimatic conditions of soda lakes, evaporite settings, and potentially soda oceans of early Earth and extraterrestrial planets.

## Abstract 2

We present a comprehensive methodology, with a case study of a saline soda lake, applicable for monitoring evaporitic mineral precipitation and hydrochemical evolution of natural brines. The significance of the synergy of multiple methods in understanding mineral assemblages, precipitation sequences, and textural relations of modern and ancient evaporite deposits is discussed.

## Introduction

Crystal nucleation and growth usually proceed between narrow compositional or thermal limits and produce a material record of these growth conditions in the form of crystals of a given phase with a given size, morphology, texture, and patterns. In material sciences, we select the growth conditions to produce crystals with some predefined properties. In earth sciences, we can go in the reverse direction, trying to determine the growth conditions from the properties of natural crystals. This strategy has been previously discussed^1,2^ and used, for instance, to constrain the growth conditions of the unique, giant gypsum crystals in Naica, Mexico,^3,4^ growth mechanisms of magnesium and sodium salts of playa lakes,^5^ or the chemistry of sea 3.5 billion years ago.^6^ This crystal growth “reverse engineering” requires a good knowledge of the crystallization processes, typically based on thermodynamic relations between phases. Crystallization of highly soluble salts from evaporating natural brines implies high concentration, chemically complex solutions from which time-dependent, kinetically controlled processes lead to the crystallization of evaporitic minerals. Consequently, an experimental investigation of the crystallization sequence is needed to complement thermodynamic modeling. This would allow the use of evaporites as proxies for the study of ancient water bodies, the paleoclimatology of basins in past arid climates, and the early stages of continental breakup during the movement of tectonic plates.^7^

Crystallization of evaporites from present-day seawater is relatively well understood, but the sequence of minerals crystallizing in closed hydrological system depends on the lithologies leached by the waters contributing to the lake.^8−10^ This variability has been roughly classified in five major water types^8^ that do not allow the accurate prediction of detailed mineralogical sequences with textural, size, morphological, and pattern information. In this work, we present a methodology for in situ studies of sequential evaporation/crystallization of brines from soda lakes in the African Rift Valley. The knowledge of the mineral precipitation sequence in saline and soda lakes is crucial to understanding the hydrochemical evolution of brines, paleoenvironmental conditions (e.g., temperature, pCO_2_) of the evaporite deposits in the geological record, scaling in geothermal power facilities, and implications for industrial crystallization of these materials.^11−17^ The assemblage of sodium carbonate minerals precipitated from soda brines are also important for constraining the geochemical conditions of soda oceans in Precambrian Earth, when life is thought to have originated, and other Earth-like planets.^18,19^

Lake Magadi is a saline pan where mainly trona, thermonatrite, and halite precipitate during the dry seasons. Since 1911, soda ash and common salt has been mined by precipitating trona and halite respectively via solar evaporation of the lake brines in artificial pans with further industrial processing.^20^ The hypersaline alkaline brines harbor a unique biodiversity relevant to the study of physiological adaption of extremophiles^21^ and their biotechnological applications.^22,23^ Trace fossils of organisms from the precursor lake of Magadi are also key to understanding depositional environments.^24^

Owing to their scientific and economic values, much work has been carried out on the mineral precipitation in soda lakes based either on field data or on thermodynamic modeling.^15,25−27^ Some recent works have proved that a combination of field research, lab experiments, and computer modeling is key to understanding evaporitic brine evolution and mineral precipitation sequences.^16,28,29^ So far, there are no experimental investigations on the mineral precipitation sequence in soda lakes. In this work, we present a comprehensive experimental investigation and geochemical modeling of the mineral precipitation sequence and hydrochemical evolution of Lake Magadi brines, using evaporative mineral precipitation experiments monitored by (a) in situ video microscopy and synchrotron X-ray diffraction from acoustically levitated droplets; (b) ex situ Raman spectroscopy, X-ray diffraction, and scanning electron microscopy; and (c) computational modeling.

## Experimental Section

### Solution Chemistry

Brine samples were collected from Lake Magadi during the field campaign in March 2018. All evaporation and precipitation experiments reported in this work use one sample selected for having a composition close to the average of Magadi waters (sampling point located at 36.27° E and 1.84° S, 605 m above sea level). The pH (±0.02), temperature (±0.15 °C), total dissolved solids (TDS; ±1 mg/L), electrical conductivity (EC; ±1 μS/cm), oxidation/reduction potential (ORP; ±1 mV), and saturation percent of dissolved oxygen (DO; ±1%) were measured in situ at the sampling time with a Hanna HI 9829 multiparametric probe.

CO_3_^2–^ and HCO_3_^–^ concentrations in the samples were determined by titration with sulfuric acid using methyl orange and phenolphthalein as indicators at the Laboratorio de Estudios Crystalográficos (LEC) of the Instituto Andaluz de Ciencias de la Tierra (IACT) in Granada (Spain). SiO_2_ was analyzed by using inductively coupled plasma optical emission spectrometry at the Technical Services of the Estación Experimental del Zaidín (CSIC) in Granada (Spain). All the remaining chemical analyses were performed by ALS Laboratory Group SL: Na^+^, K^+^, Mg^2+^, Ca^2+^, Al^3+^, Fe (total), Ba^2+^, B^+^, Sr^2+^, Br^–^, and I^–^ were determined by using inductively coupled plasma mass spectrometry (ICP-MS). The samples were fixed by the addition of nitric acid prior to analysis with ICP-MS. Cl^–^, F^–^, and SO_4_^2–^ were analyzed by using ion liquid chromatography. PO_4_^3–^ was determined by a colorimetric method based on molybdenum blue using discrete spectrophotometry.

Table 1 shows the in situ and laboratory measurements of the hydrochemical parameters of the Lake Magadi sample used for the evaporation experiments. The dominant ions are Na^+^, K^+^, CO_3_^2–^, HCO_3_^–^, and Cl^–^ with relatively high levels of SO_4_^2–^, F^–^, SiO_2_, PO_4_^3–^, and Br^–^. However, these brines are depleted in Ca^2+^, Mg^2+^, Al^3+^, and Fe.

**Table 1:** Results of the In Situ Brine Characterization and the Chemical Analysis of the Sample Used for the Evaporation and Precipitation Experiments^a^

in situ pH	*T* (°C)	EC (mS/cm)	TDS (g/L)	ORP (mV)	DO (%)	ionic strength (M)
9.8	29.2	68.6	38.1	–464.7	46.2	0.74

Na^+^	K^+^	Cl^–^	CO_3_^2–^	HCO_3_^–^	SO_4_^2–^	SiO_2_	F^–^	PO_4_^3–^
12850 ± 1285	228.5 ± 23	5250 ± 788	9600	6100	100.5 ± 15	91.4	208 ± 31	19.4 ± 3.9

Mg^2+^	Ca^2+^	Al^3+^	Fe^tot^	B^+^	Ba^2+^	Br^–^	Sr^2+^	I^–^
12 ± 0.12	4.5 ± 0.45	<0.05	0.1 ± 0.01	5.2 ± 0.52	0.03 ± 0.003	23.3 ± 2.3	0.04 ± 0.004	0.8 ± 0.08

a Ionic concentrations are in ppm.

### Evaporation Experiments

Evaporation and mineral precipitation experiments were performed at 23 ± 1 °C on glass slides. Droplets of about 25 μL (approximate diameter of 1 cm) were poured on glass slides using plastic pipets. Two series of evaporation experiments were implemented: in the first one, droplets were left to evaporate until complete desiccation while in the second the experiment was stopped after the initial stages of precipitation, removing all the liquid with highly absorbent paper to ease the identification of the first precipitates occurring in small amounts. Both series were monitored with optical video microscopy (Nikon AZ100).

### Ex Situ Characterization of Precipitates

The mineralogy of the precipitates was characterized after evaporation experiments with the use of X-ray diffraction, Raman spectroscopy, and electron microscopy. For ex situ X-ray diffraction we used a high-resolution Bruker D8 Advance X-ray diffractometer (at the Laboratorio de Estudios Crystalográficos) with monochromatic Cu Kα_1_ radiation, primary Ge(111) monochromator, and a Lynxeye PSD detector. Diffractograms were acquired in transmission mode, at 40 kV acceleration voltage and 40 mA current, with 2θ scans spanning from 5 to 80° with a 2θ step of 0.02° s^–1^. Malvern Panalytical HighScore software (version 4.9) with the ICCD PDF-4+ (2020) database was used for phase identification. Raman spectra were recorded by using a HORIBA Jobin Yvon LabRAM high-resolution Raman spectrometer equipped with an Olympus BX41 optical binocular microscope with Koehler illumination and a CCD detector. An excitation beam with a wavelength of 532 nm (frequency-doubled neodymium-doped yttrium aluminum garnet laser) and output power of 100 mW was used. Spectra were acquired with an exposure time of 5–10 s and accumulation of 10–60 times to improve the signal-to-noise ratio. Electron microscopy was used to characterize the texture and the local elemental composition of the precipitates with a Zeiss Supra 40VP field-emission scanning electron microscope (FESEM) equipped with an Oxford energy-dispersive X-ray analyzer (EDX) at the Centro de Instrumentación Científica (CIC) of the University of Granada (Spain) operating at 5–20 keV. After complete desiccation, glass slides containing the precipitates were directly mounted on the SEM stub by using double-faced conductive carbon tape for electron microscopic analysis.

### In Situ Characterization of Precipitates

In situ X-ray powder diffraction data was collected from evaporating, levitated droplets to track the time evolution of mineral precipitation during evaporation. These experiments were performed at the μSpot beamline (see ref (30) for details) at the BESSY II synchrotron (Helmholtz Centre Berlin for Materials and Energy, Berlin, Germany), using the protocol described in ref (31). The beamline features an acoustic levitator used as a containerless sample holder (see the experimental setup in Figure 10a). In a typical experiment, 5 μL of the sample was pipetted in one of the nodes of the standing acoustic wave of the levitator to form a drop with an approximate diameter of 2 mm. The droplets were maintained into the beam during data collection by gradually lowering the reflector of the acoustic wave with decreasing volume of the droplets. An incident beam having a 0.72929 Å wavelength and a 100 μm size was used. Scattered intensities were collected by a two-dimensional detector (Eiger9M, CCD 3072 × 3072) for 5 s exposures. Evaporation was followed for 80 min until complete desiccation. The evolution of the size of the levitated droplet during evaporation was monitored with a video camera. Levitated drop evaporation was performed at 25 ± 1 °C and relative humidity of 35 ± 2%.

### Computer Modeling

The PHREEQC version 3.4 code with the Pitzer database was used for thermodynamic hydrochemical speciation, evaporation, and precipitation calculations.^32^ In a first step, evaporation was simulated at 25 °C to calculate the saturation index of all mineral phases in the database during the evaporative concentration of the brine. In a second step, we calculated the equilibrium crystallization of all phases that were supersaturated during the first step. The crystallization of minor phases, and the inhibition of phosphate precipitation (due to Ca deficit) has been addressed by our thermodynamic calculations by supplementing the PHREEQC Pitzer database with fluorite, hydroxyapatite, and fluorapatite taken from the llnl.dat database^32^ and chlorapatite from the Thermoddem database (version 1.10).^33^ For villiaumite (NaF), thermodynamic data was taken from the ThermoChimie database (version 10a).^34^ Parameters for Na(H_2_PO_4_) and its hydrated forms were taken from ref (19).

## Results

**Figure 1:**
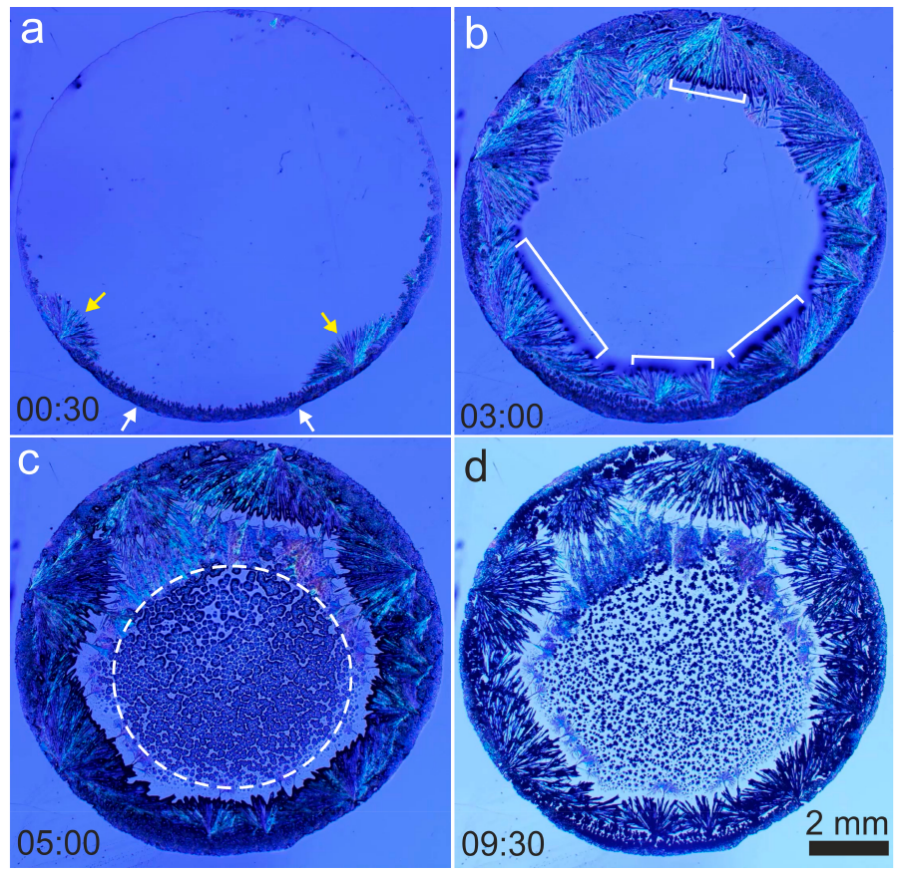
Overall mineral crystallization sequences and patterns formed during the evaporation of a droplet. White arrows, the first dendritic minerals; yellow arrows, thick acicular crystals that form at the second stage; square brackets, crystals that form at the third stage as dark spots. The full sequence is presented in Video S1. Time (bottom left corner of each picture) counts from the beginning of precipitation.

Crystallization during the evaporation of the droplet follows a characteristic sequence and produces distinct mineral patterns. Figure 1 shows the overall sequence of crystallization processes during the evaporation of the drop (the full sequence is presented in Video S1). Mineral precipitation starts on the border of the droplet as dendritic crystals (Figure 1a, white arrows) followed by bunches of thick acicular crystals that nucleate at the tips of the dendrites and grow toward the center of the droplet (Figure 1a (yellow arrows) and 1b). At the center of the drop, within the dashed circle in Figure 1c, precipitates form after the thick acicular crystals have stopped growing (Figure 1c). Crystallization at the center continues until complete desiccation of the brine there, but some liquid is still entrapped between crystals of the external rim, producing the latest precipitation events in the sequence (Figure 1d). The initial crystallization on and near the edge of the drops could be due to pinning of the contact line on the hydrophilic glass surfaces and the outward capillary flow of solutes from the center during evaporation, creating chemical gradients. The wetting property of the glass surface and the emerging crystals, and crystallization-driven flow of solutes may control the subsequent patterns after initial nucleation.^35,36^ A relatively higher evaporation rate seems responsible for the patterns observed on the center (Figure 1d), creating tiny outward radiating crystals.

**Figure 2:**
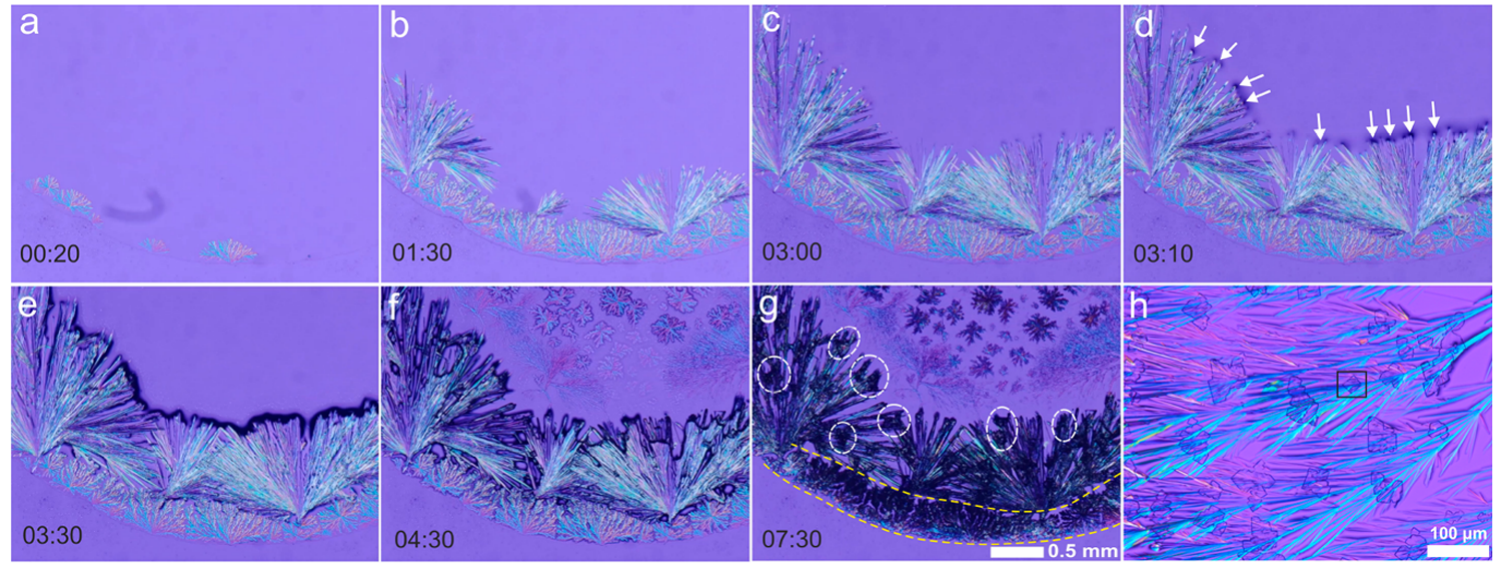
Frames from Video S2 (acquired at the indicated time in minutes since the precipitation began) showing the details of the precipitation sequence of the border dendrites, the acicular minerals, and the irregular dark spots (arrows in panel d and circles in panel g) forming between the acicular minerals. Panel h is a close-up view of the irregular dark spots in panel g showing the aggregates of cubic crystals (note the single cubic crystal inside the black square).

**Figure 3:**
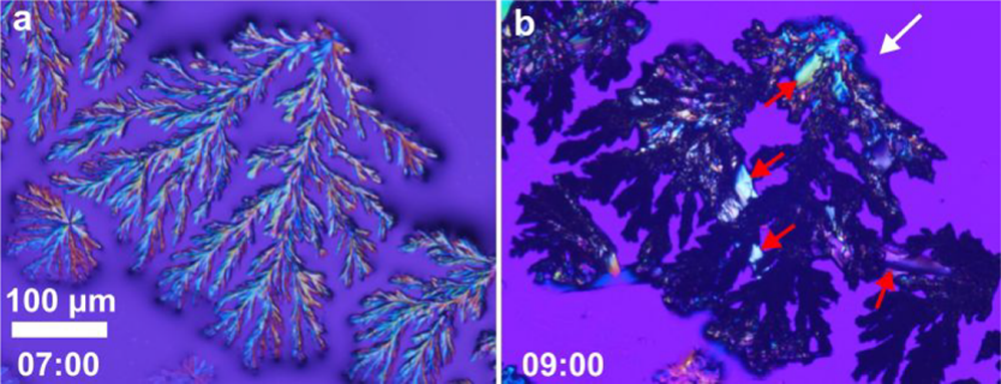
Evolution of border dendrites area during last phases of the precipitation sequence that took place between the branches. The white arrow points from the border to the center of the droplet; red arrows show transparent bladelike crystals beneath the border dendrites.

Figure 2 (full sequence in Video S2) shows the details of the precipitation at the border. As shown in Figure 2a and Video S2 (blue arrows), the precipitation process begins on the border of the droplets in the form of dendritic crystals. These dendrites start to precipitate on the liquid/air/glass interface of the droplet, which was clearly observable from top-view images. After the dendrites, bunches of acicular crystals nucleate and grow toward the center of the droplet while spreading sideways (see Figure 2b,c and Video S2 (blue arrows)). Figure 2c corresponds to the end of the precipitation of the thick acicular crystals and the beginning of a third mineral pattern in the form of irregular dark spots. These irregular-shaped mineral grains are shown in Figure 2d (white arrows) and 2e. Once the irregular-shaped minerals began to form, precipitation at the center of the drop starts (described in the next paragraph). From this time on, the desiccation and precipitation processes in the border advance in a reverse direction (toward the border of the droplet) where the acicular crystals and the border dendrites were already present (see Figure 2f,g and Video S2 (green arrows)). The white dashed ellipses in Figure 2g show the irregular-shaped grains that precipitate between the acicular crystals. High-resolution micrographs show that these irregular dark spots are aggregates of well-developed cubic crystals (see Figure 2h), most probably halite. The last phase of the precipitation process took place beneath the border dendrites as shown in Figure 2g (between the yellow lines). Figure 3 and Video S3 show the details of the precipitation process in this area. Figure 3a shows the area where the first dendrites formed. The transparent bladelike crystals (indicated by the red arrows in Figure 3b) precipitate from the residual water below the dendrites at the end of the evaporation process. In addition, the first precipitated dendrites (Figure 3a) change from transparent to opaque because of the latest precipitation of other minerals from capillary water entrapped between the dendrites. In qualitative terms, the precipitation rate of the minerals from the residual brine held between the dendritic and the acicular trona was the slowest whereas the fastest precipitation rate was observed on the center. The second and third faster rates of precipitation were observed with the dendritic and acicular crystals of trona, respectively.

**Figure 4:**
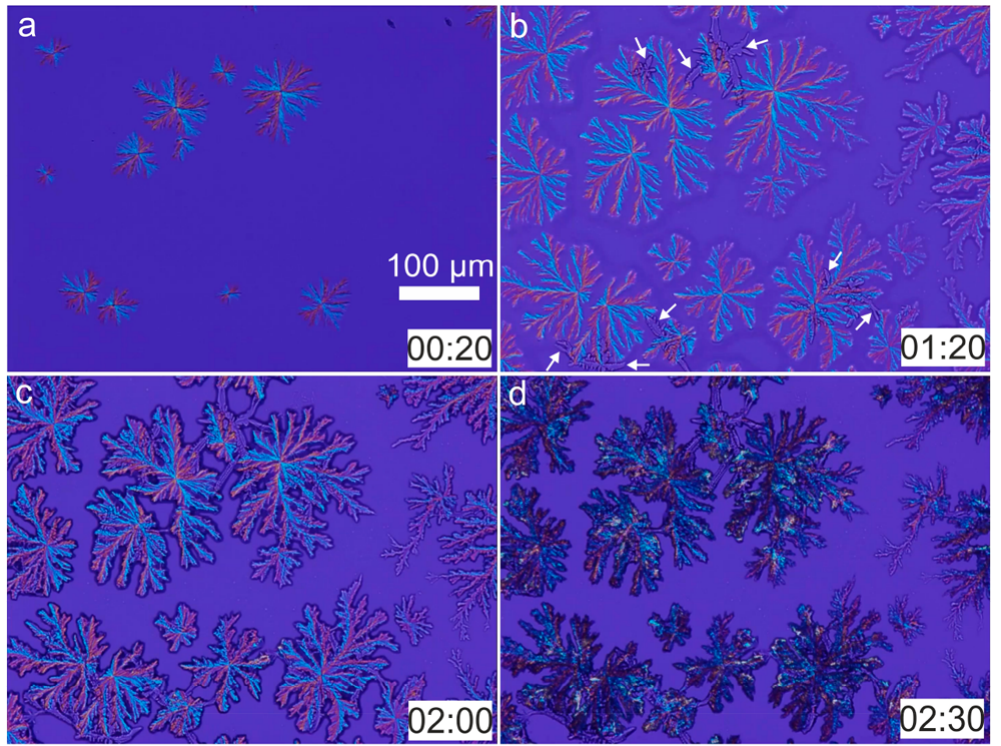
Frames taken from Video S4 showing the precipitation process and mineral patterns on the center of the droplets. The white arrows indicate the crystals forming after the starlike dendritic minerals.

Figure 4 (full sequence in Video S4) shows the details of the crystallization sequence at the center of the droplets. These precipitates appear later than the cubic crystals on the tips of the acicular crystals (see Figure 2d). Precipitation at the center begins with starlike dendritic minerals that radiate from the center outward (Figure 4a). Once the precipitation of the radiating branches ceases, other crystals (also dendritic) start to precipitate between their branches as shown by the white arrows in Figure 4b. Finally, the residual brine entrapped between the branches of the dendrites evaporates, producing a later precipitate on the preexisting dendrites that darken them (Figure 4c,d).

**Figure 5:**
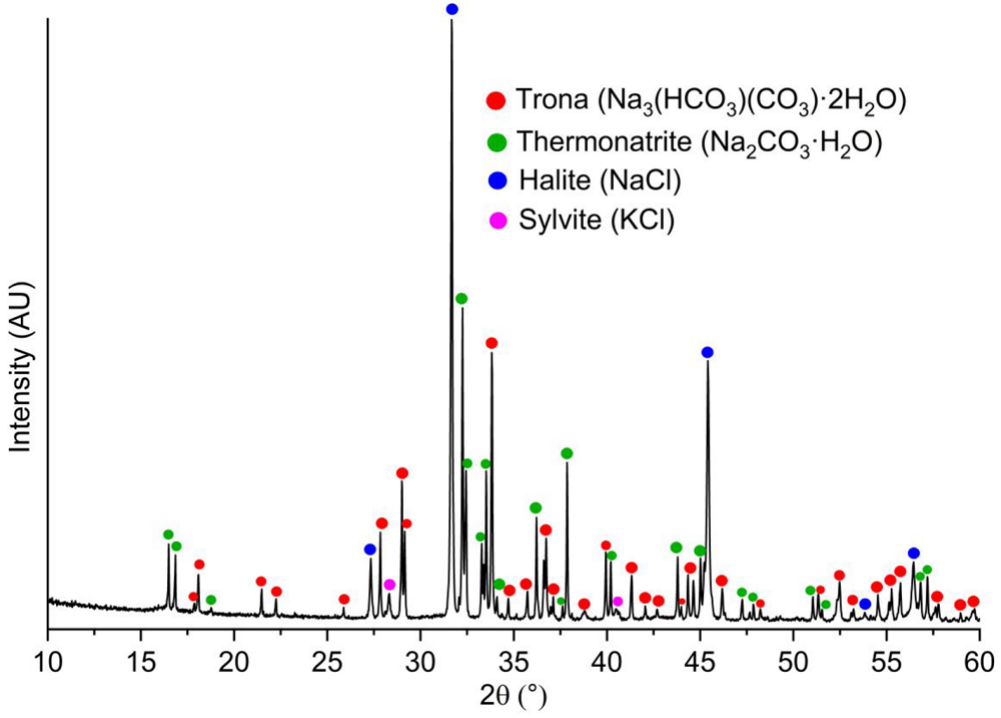
Powder X-ray diffraction patterns of whole precipitates.

The minerals making these patterns were identified by using X-ray diffraction and Raman spectroscopy. Powder X-ray diffraction of the whole precipitates from dry droplets revealed the presence of trona, thermonatrite, halite, and minor sylvite (Figure 5).

**Figure 6:**
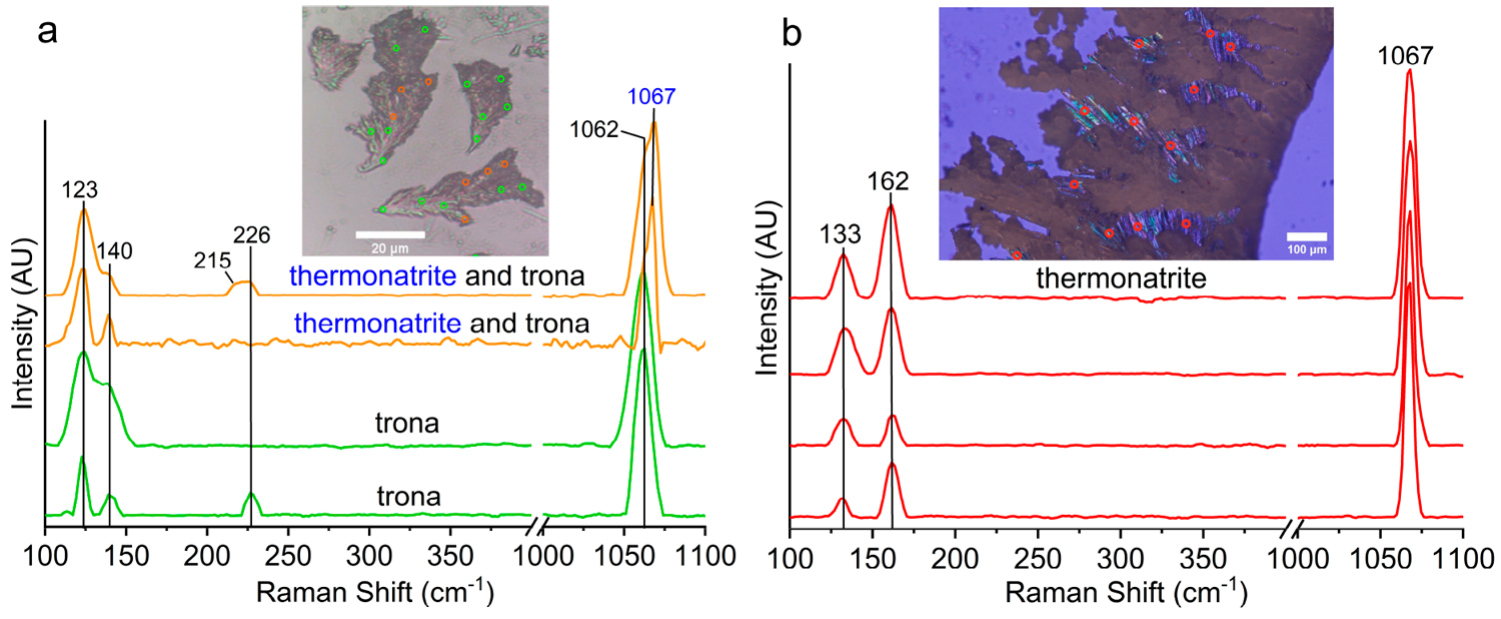
Raman spectra of (a) border branches that form first (green dots, trona; orange dots, trona and thermonatrite) and (b) the last precipitates that form beneath and between the border trona branches (red dots, thermonatrite).

Raman spectra of the border dendrites that form first were collected after gentle drying of the drop with absorbent paper to avoid further precipitation. These spectra indicate that these dendrites are made of trona (Figure 6a). In addition, some thermonatrite was detected probably from the evaporation of the water that could not be completely removed. Raman spectra of the last minerals of the precipitation sequence that form beneath and between the dendritic trona (Figure 3 and Video S3) revealed thermonatrite (Figure 6b). The microscopic image in Figure 6a was taken after removal of the water with filter paper, whereas the one in Figure 6b was taken after complete evaporation of the whole solution in the border. The Raman modes between 100 and 300 cm^–1^ are assigned to the lattice modes, whereas the vibration peaks at 1062 and 1067/1068 cm^–1^ represent the symmetric stretching modes of carbonate groups in trona and thermonatrite, respectively.^37,38^

**Figure 7:**
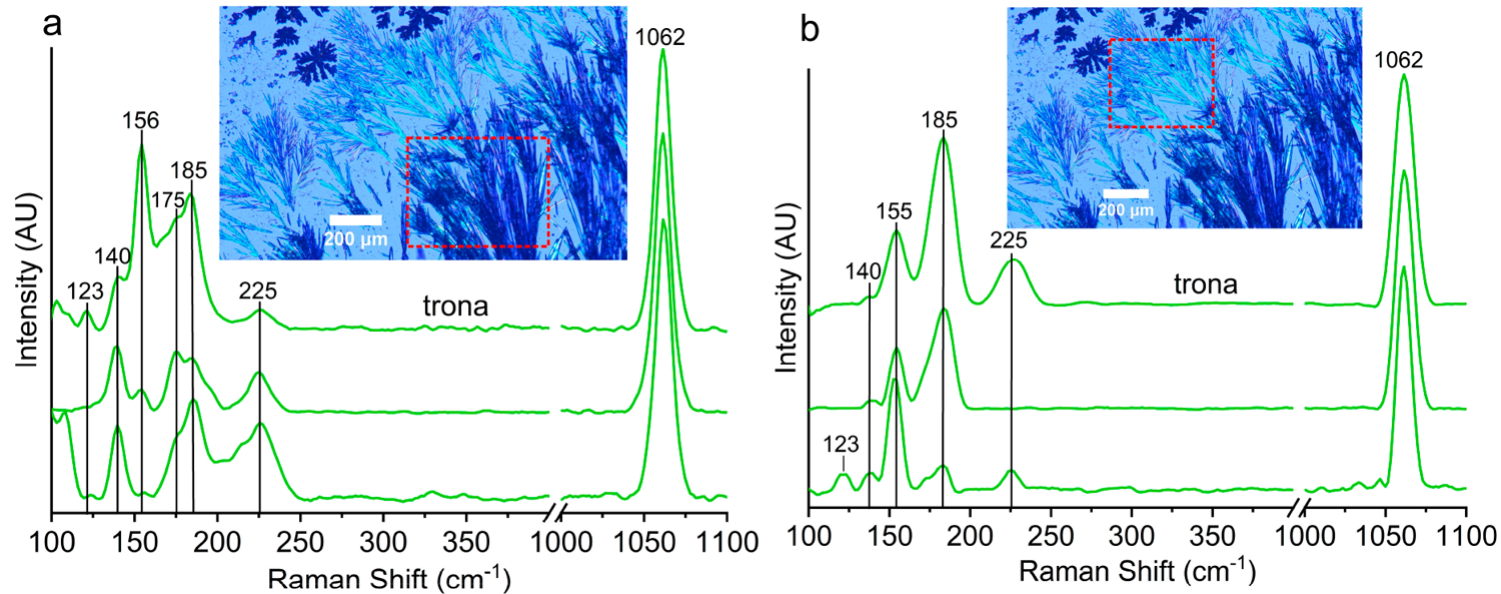
Raman spectra of (a) thick and (b) thin acicular minerals that form next to the border dendrites.

The second type of crystals that precipitate next to the border trona dendrites were thick, and the next were thin, acicular crystals (Figure 2a–g and Video S2). Raman spectra of these acicular crystals show that they are made only of trona (Figure 7). Once the growth of the acicular crystals ceases, aggregates of cubic crystals start to crystallize (Figure 2g (dark spots) and 2h). These cubic crystal aggregates show no Raman signal, suggesting halite or villiaumite.

**Figure 8:**
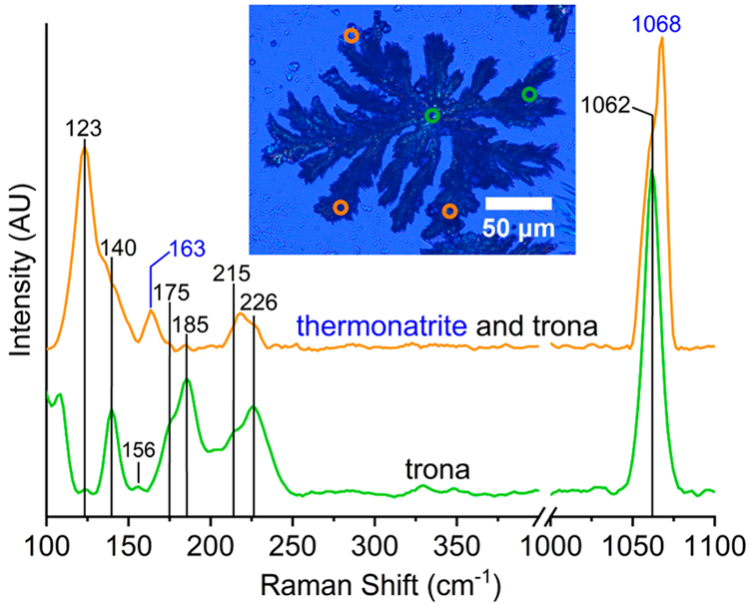
Raman spectra of radiating starlike minerals that form at the center of the droplets (green dots, trona; orange dots, trona + thermonatrite).

Raman spectroscopy of the central dendritic minerals revealed a combination of trona and thermonatrite (Figure 8). The crystallization of these dendrites has similarities with the initial crystallization at the border of the droplet. This implies that the first mineral that forms at the center could be trona, whereas thermonatrite comes later due to the later desiccation of the residual water held in the pores between trona crystals.

**Figure 9:**
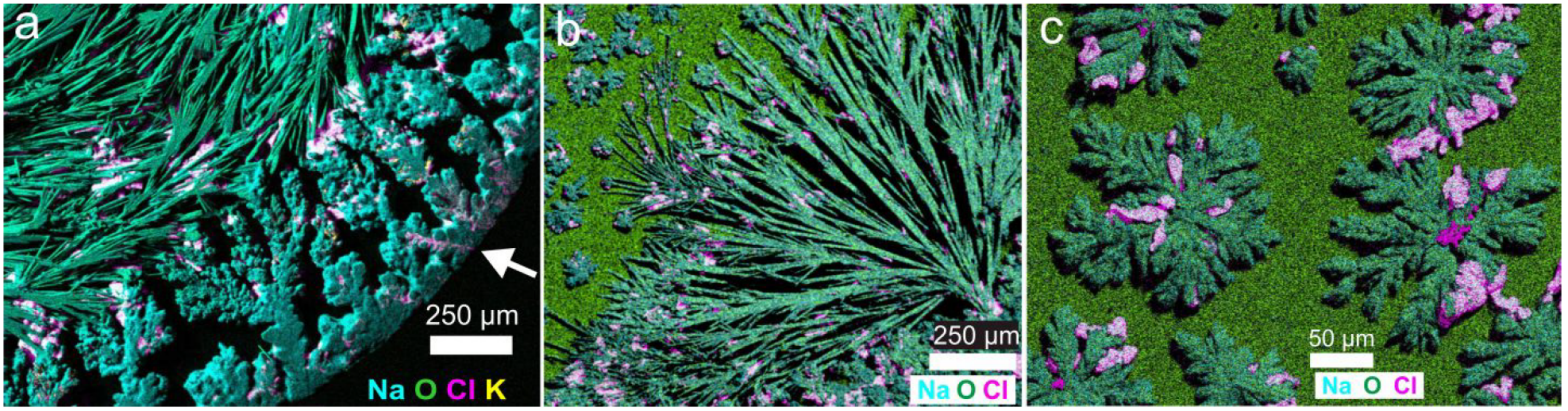
EDX map of (a) border precipitates (white arrow points from the border to the center of the droplet), (b) acicular minerals together with irregular aggregates, and (c) radiating starlike minerals that form on the center.

**Figure 10:**
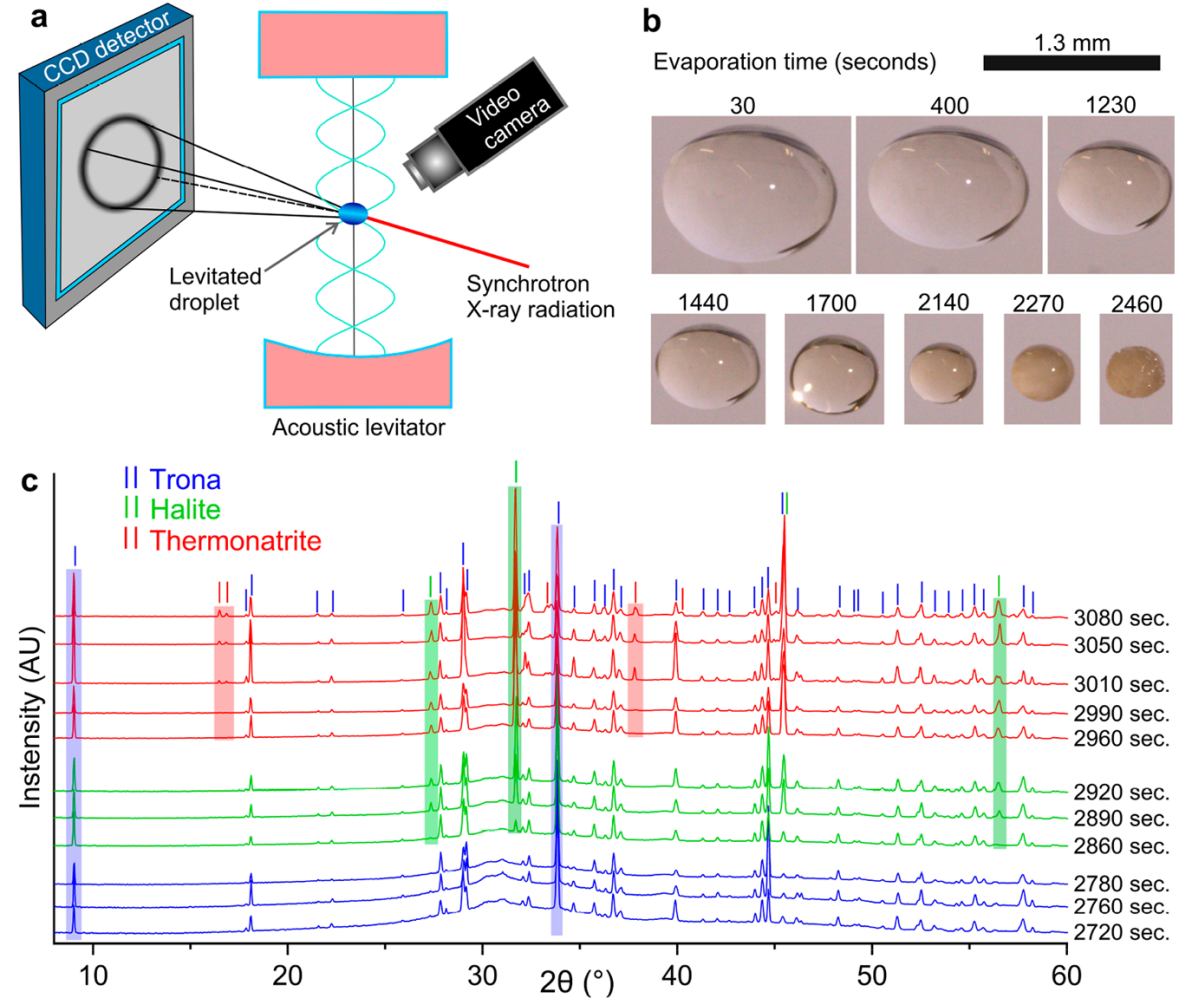
(a) Experimental setup used for in situ X-ray diffraction measurements from levitated drops. (b) Change in the size of the drop during evaporation. (c) X-ray diffractograms collected during the evaporation of levitated droplets of brine. Note that the first diffractograms of the corresponding color are taken a few seconds after precipitation of the corresponding mineral begins so that the characteristic peaks of the given phase are clearly visible. The exact time when each mineral starts to precipitate is reported in the text.

The distribution of mineral phases and the presence of minor or amorphous precipitates were tested by using EDX elemental composition maps of the precipitates that form on different parts of the droplet. These maps show mainly Na–CO_3_ minerals, NaCl (halite), and minor KCl (sylvite) and NaF (villiaumite) (Figure 9, Figures S2 and S3). Figure 9a shows the border precipitates. Raman spectra revealed that these Na–CO_3_ minerals are trona and thermonatrite (Figure 6). Halite and sylvite, which are Raman inactive minerals, were identified by EDX analysis. SEM backscattered electron images show that the border dendrites were highly feathery (Figure S1a). The needlelike crystals are trona crystals with smaller halite crystals between them (Figures 2g,h and 9a, and Video S2). These halite crystals were the second mineral in the precipitation sequence (Figure 10b). Semirounded cubic crystals of villiaumite have been observed between the dendritic trona crystals (see Figures S2 and S3). The dendrites at the center are Na–CO_3_ minerals (trona and thermonatrite) with halite (Figure 9c). Once the precipitation of the trona dendrites ceases, halite and villiaumite precipitate between their branches (Figure 4b and Video S4). The crystals pointed out by the white arrows in Figure 4b were halite as revealed by the EDX maps (Figure 9c). The dendrites are open-textured aggregates like the initial dendrites at the border (see Figure S1c).

In situ, containerless X-ray diffraction data from evaporating, levitated droplets were collected in the μSpot beamline at BESSY to check for any effect related to the glass substrate or the flat geometry of the drying drop (see Figure 10a). Figure 10b shows eight successive pictures of the levitated drop recorded to track the time evolution of the droplet volume during evaporation. Figure 10c shows the in situ X-ray diffractograms collected during evaporation. Trona precipitates first after 2670 s since evaporation began. Precipitation of halite began 3 min later (after 2855 s; see the green bars). Finally, precipitation of thermonatrite started after 2955 s (red bars). The in situ X-ray diffraction data confirmed the precipitation sequence that was observed in the previously described experiments.

**Figure 11:**
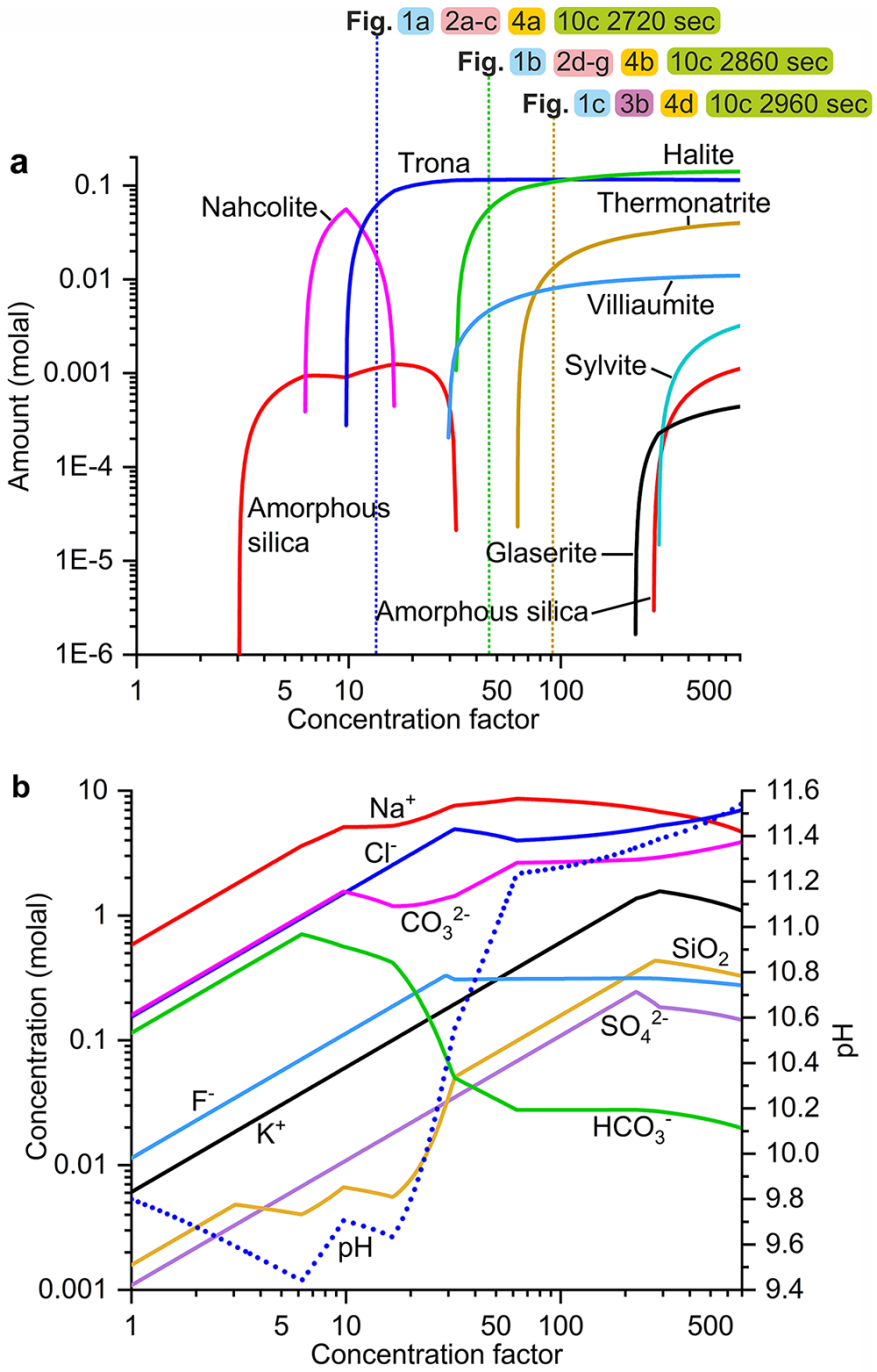
PHREEQC simulation of evaporation and mineral precipitation from Lake Magadi brine at 25 °C: (a) the mineral precipitation sequence and the amount of precipitate and (b) chemical evolution of the brine during mineral precipitation. In panel a, three key points during evaporation are marked by vertical dotted lines labeled with references to previous figures where the status of the brine at this point is shown. Blue, green, and yellow dotted lines represent the time when trona, halite, and thermonatrite precipitation is dominant during the sequence.

Most previous knowledge on the precipitation sequence from soda lake brines comes from thermodynamic modeling.^15,25−27^ To set our results in this framework, and to check for any unexpected behavior, mostly large kinetic effects, we have performed a set of hydrochemical modeling runs using the PHREEQC code. These simulations use Pitzer speciation^32^ using the pitzer.dat database provided by PHREEQC 3.4 after inclusion of the missing species (alkalinity, Ca, Mg, K, S, Si, and F) and the corresponding speciation and dissolution reactions from different databases as mentioned in the Experimental Section. Evaporation and mineral precipitation simulations compute successive equilibrium states separated by discrete steps of slight water removal. Figure 11 shows the output of this model in terms of the sequence of mineral crystallization (a) and ionic concentration of the relevant species (b) versus concentration factor (CF; the ratio between the initial brine volume and the current volume at each time). The output of the model reproduces very well the experimental observations.

Among the phases identified by in situ and ex situ experiments, trona was predicted to precipitate first at a CF of 9.7, which agrees with the experimental observations. Evaporation linearly raises the concentrations of Na^+^ and CO_3_^2–^ until the precipitation of trona commences (Figure 11b). Once trona precipitation sets in, sodium and carbonate species start to deplete until the CF reaches a value of 16. Nahcolite is predicted to precipitate for a short period between CFs of 6.2 and 16.4, depleting the HCO_3_^–^ concentration. This depletion continues due to the onset of trona precipitation via the dissolution of nahcolite. After a CF of 30, trona no longer precipitates due to the minimal HCO_3_^–^ concentration, leaving a constant amount of about 114 mmol/kg. However, nahcolite has not been identified in the lab experiments, due to either a short transient presence in the drops or to slow nucleation kinetics.

Halite and villiaumite are predicted to precipitate in the second phase (Figure 11a). The amount of precipitated halite was predicted to be larger than the amount of trona after complete desiccation. Villiaumite precipitates at a CF of 30, almost simultaneously with halite which starts forming at a CF of 32. Thermonatrite crystallization is predicted at a CF of 62.6. Sylvite and glaserite appear near the complete dryness of the brine due to K^+^ and SO_4_^2–^ evaporative concentrations (Figure 11b). The pH evolution during evaporation was controlled by the CO_3_^2–^/HCO_3_^–^ ratio. Initially, pH decreases slightly from 9.8 to 9.4 until a CF of 6.4 (Figure 11b, blue dotted line). Afterward, pH steeply rises until a CF of 63 due to the steep decline in HCO_3_^–^ concentration following the subsequent onset of nahcolite and trona precipitation. Once thermonatrite precipitation set in (after a CF of 63), pH rises gently with CO_3_^2–^ concentration.

On the basis of the thermodynamic model, the initial Lake Magadi brine was supersaturated with respect to fluorapatite and magnesite (see Figure S4a). The amounts of fluorapatite and magnesite predicted to precipitate are about 0.02 and 0.5 mmol, respectively. These minerals were not detected in the lab experiments likely because of their minor amounts. Simulation of evaporation in the absence of fluoride and phosphate ions shows that the initial Lake Magadi brine was supersaturated with respect to calcite and magnesite (see Figure S5a). At a CF of 8.6, calcite starts dissolving and gaylussite begins to form until a CF of 11.5. Pirssonite came to equilibrium with the solution after a CF of 11.5 via dissolution of gaylussite. However, calcite, magnesite, gaylussite, and pirssonite were not detected in the lab experiments most likely because of their trace amounts in the mix of minerals. The amounts of calcite, gaylussite, and pirssonite predicted to precipitate are about 0.12 mmol, whereas that of magnesite was 0.5 mmol. As a result of their trace amounts, precipitation of Ca–Mg carbonate minerals did not deplete the carbonate ions during evaporation simulation (see Figure S5b).

Amorphous silica supersaturates at a CF of 3 (see Figure 11a), reaching a maximum amount of precipitate (1.2 mmol/kg of brine) at a CF of 17. From this point on, it began to redissolve because of the steep pH rise that increases the solubility of SiO_2_.^39^ Amorphous silica starts to precipitate again when the brine is about to dry, after a CF of 275.

## Discussion

Deriving conclusions on the natural evaporitic crystallization in Lake Magadi from these drop evaporation experiments requires scaling from the millimeter to the tens of meters spatial scale and from the minutes to the year (seasons) temporal scale. After this scaling, the experimentally observed evaporative precipitation sequence explains many features of the Magadi area deposits and the current precipitation in the lake. Trona has precipitated in Lake Magadi for the past 100 000 years and continues today, forming 65 m thick evaporite deposits.^40^ Precipitation occurs at both the bottom and the surface of the lake. Long vertical bladed crystals with sharp points grow upward from the bottom of the water body as stellate groups, forming a firm mesh of crystals. Thin trona crusts precipitate on the water surface^15,41−43^ as aggregates of smaller crystals growing at higher supersaturation values under fast evaporation. The experimentally observed initial phase producing dendritic trona and bunches of fast-growing trona needles on the border of the droplets corresponds to the growth of vertical bladed crystals from the bottom of the lake. The third phase of high supersaturation thermonatrite precipitation in the central region of the drop, concomitant with the latest precipitation of trona and halite, is equivalent to the precipitation of thin crusts at the surface of the lake. The upward radiating blades terminate against the trona crust on the surface, which serves as the nuclei of the following generation of crystals for the next year. The successive growth of crystals in this manner gives rise to a layered appearance of trona deposits,^15,41−43^ made of 2–5 cm annual bands^41^ with a content of trona of about 90%.^44^ The precipitation of thermonatrite and halite between the dendritic and acicular trona crystals in lab experiments is relevant for the interpretation of the Magadi evaporitic sequence. Thermonatrite and halite form only in the modern evaporite sequence but are not reported in the ancient deposits of Lake Magadi. This must be due to a change in the climatic/hydrologic conditions of the lake; either the paleolake brines did not reach supersaturation required for these minerals to precipitate or they precipitated but redissolved during an episodic freshening of the paleolake brines. In contrast to the natural lake, significant amounts of thermonatrite and halite precipitate respectively at the bottom and on the surface of the artificial pans of TATA Chemicals made for the commercial production of salt by harvesting the top layer of halite.^42,45^ The precipitation of significant amounts of thermonatrite and halite in these pans is explained by our results: the company pumps the HCO_3_-depleted lake brine (after trona precipitation) into shallow evaporation ponds where evaporation is faster than in the lake, fresher water inputs are absent, and organic production of CO_2_ is negligible. Fast evaporation in these HCO_3_-depleted ponds, as in the last stage of our experiments, precipitates large amounts of thermonatrite and halite with some trona.^42,45^

Drill cores of the evaporite series also contain nahcolite.^40,41^ In our calculations, nahcolite is predicted to appear early during evaporation and then redissolve, but it was not identified during the laboratory experiments. This can be due to kinetic effects, not included in our calculations because no data is available on this kinetics; nahcolite precipitation should be slow with respect to drop evaporation speed. Nahcolite primarily precipitates on the bottom of Nasikie Engida, where there is a higher flux of magmatic CO_2_ along the fault zones.^15,46^ The opposite trend of the CO_3_^2–^/HCO_3_^–^ ratio after a CF of 15 reflects the precipitation of trona that is limited by the low HCO_3_^–^ content of the brine. The later increase in CO_3_^2–^ and decrease in HCO_3_^–^, and the corresponding increase in pH, lead to the precipitation of thermonatrite and the end of trona precipitation.

The early precipitation of calcium carbonate minerals produces the depletion of calcium, which results in a lack of apatite precipitation^19,47^ and the consequent accumulation of phosphate in soda lakes up to levels relevant to the syntheses of prebiotic biomolecules. Lake Magadi samples were supersaturated with respect to fluorapatite since the beginning of evaporation. The amount of fluorapatite was limited by the very low calcium content. As a result, phosphate concentration increases linearly with evaporation (see Figure S4a,b). Apatite precipitation has not been reported in Lake Magadi because of the absence of or a minor amount of Ca in the brines. In the simulation, phosphate concentration was overestimated due to microbial phosphate consumption and deposition with organic matter in the actual environment.^19^ Based on the model predictions, there were no Na(H_2_PO_4_) mineral precipitations until almost complete dryness.

Other consequences of the depletion of Ca in the brines are the lack of fluorite precipitation and the evaporative accumulation of fluoride until villiaumite supersaturation. Once villiaumite precipitation begins at a CF of 30, the fluoride content equilibrates. Villiaumite is a common constituent of the surface deposits of Lake Magadi brines.^41,42^

Calcite, magnesite, gaylussite, and pirssonite are present as efflorescent crusts and pisolites in modern Lake Magadi and drill cores from Lake Magadi and Lake Bogoria.^15,42,48−50^ These aggregates form due to the episodic interaction of Ca-rich freshwater with the sodic brine on the lake margin.^15,40,42^ Alternatively, as observed in other saline lakes and demonstrated experimentally, Ca–Mg carbonate precipitation could have been mediated by metabolic activities in the lake water.^51−54^ Calcite spherule nucleation was observed at the sediment–water interface of highly alkaline lakes with coexisting Ca–Mg-rich hydrochemistry and microbial-driven colloidal substances, forming cemented spherulites in different depositional settings.^53^ The precipitation of these phases is predicted by our thermodynamic models in small amounts (less than 0.5 mmol/kg of brine evaporated) at the beginning of evaporation, but only in the absence of phosphate and fluoride (see Figure S5c). Precipitation of these minerals was not observed in our in situ characterization, most probably due to the very low Ca concentration of the sample, which was equilibrated with Ca phases at sampling time and lacks the additional Ca supply to the lake by later freshwater inflow or groundwater input to the lake.

In Lake Magadi, as the pH rises above 9, silica remains concentrating at the same rate as chloride due to the polymerization of silicic acid.^42,44,55^ Our thermodynamic model predicted a continuous increase in silica concentration until the initial precipitation and after redissolution of amorphous silica (from CF 6 to 32) up to close to complete dryness. In our experiments, amorphous silica has not been explicitly detected, but some fluffy precipitates can be seen in the drop earlier than trona; for example, see the last frame in Figure 10b (2460 s). However, no diffraction peaks were observed. These precipitates before trona must be amorphous phases, probably amorphous silica, representing the precipitation of siliceous phases such as opal-A, magadiite, or gels in Lake Magadi and Little Magadi.^15^ Silica gels precipitate in close proximity to the hot springs of Little Magadi lake and near the shorelines under active evaporation.^15,40,48,56^

Precipitation of sylvite and glaserite is predicted by our model close to complete desiccation, but they have not been reported in the Lake Magadi basin because complete desiccation has never or very seldomly occurred. During the rainy season, freshwater supply reaches the lake before brines reach supersaturation with respect to sylvite and glaserite. These minerals have been reported in other alkaline lakes of the East African Rift Valley, for example, in Lake Katwe (Uganda).^57^

## Conclusions

The evaporitic mineral precipitation sequence in saline and soda brines is challenging to monitor due to concomitant precipitation to many phases and the transformation between different hydrates of sodium carbonate–bicarbonate minerals and secondary mineral precipitation during sample handling under different conditions of pH, temperature, pCO_2_, and water activity. In this work, we have presented the synergy of multiple methodologies that allowed accurate determination of the mineral precipitation sequence during laboratory evaporation of Lake Magadi soda brines. The proposed combination of in situ methods used to characterize the evaporative crystallization of salts from Magadi waters shows a high potential to build and check hydrochemical models in earth sciences that can be used in (a) explaining current depositional environments, (b) interpreting the evaporitic deposits appearing in sedimentary records in terms of paleoclimatic indicators, and (c) proposing applied methods for the industrial use of these brines at the optimal time during their natural hydrochemical evolution.

The proposed methodology can be improved if additional in situ information is needed from minor, short-lived metastable or amorphous minerals.^58,59^ The most promising method for this type of study is the simultaneous use of synchrotron diffraction and Raman spectroscopy in levitated, containerless evaporation experiments. Exploring this combination will be the next step in our studies in this field.

## Supporting Information Available

Backscattered electron micrographs and elemental maps of mineral precipitates, plots showing amount of minerals predicted to precipitate and resulting hydrochemical evolution during Lake Magadi evaporation in the presence of phosphate and fluoride ions; video microscopy of overall evolution of precipitation during evaporation of a single droplet on glass slide; video microscopy of evaporation and precipitation on the border of a droplet; video microscopy of precipitation process on the border of the droplet marked with red rectangle in Video S2; video microscopy showing details of precipitation process at centers of droplets (ZIP)
