# Supplementary material for: A Comprehensive Methodology for Monitoring Evaporitic Mineral Precipitation and Hydrochemical Evolution of Saline Lakes: The Case of Lake Magadi Soda Brine (East African Rift Valley, Kenya)
Source: Cryst Growth Des. 2022 Mar 3;22(4):2307–17. doi: 10.1021/acs.cgd.1c01391 (PMC8991015; doi:10.1021/acs.cgd.1c01391)
Supplement: Supplementary file 1 — cg1c01391_si_001.zip [file cg1c01391_si_001.zip › Supporting Information.docx]

Supporting Information for

A comprehensive methodology for monitoring evaporitic mineral precipitation and hydrochemical evolution of saline lakes: the case of Lake Magadi soda brine (East African Rift Valley, Kenya)

Melese Getenet, Juan Manuel García-Ruiz, Fermín Otálora, Franziska Emmerling, Dominik Al-Sabbagh, Cristóbal Verdugo-Escamilla

a
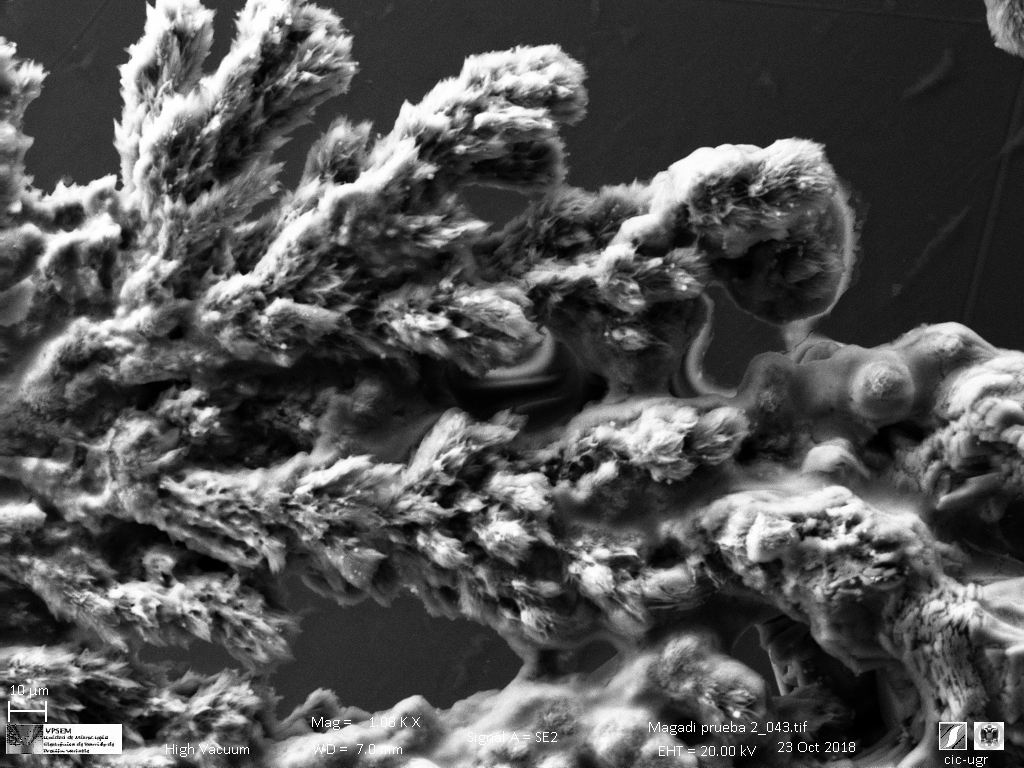

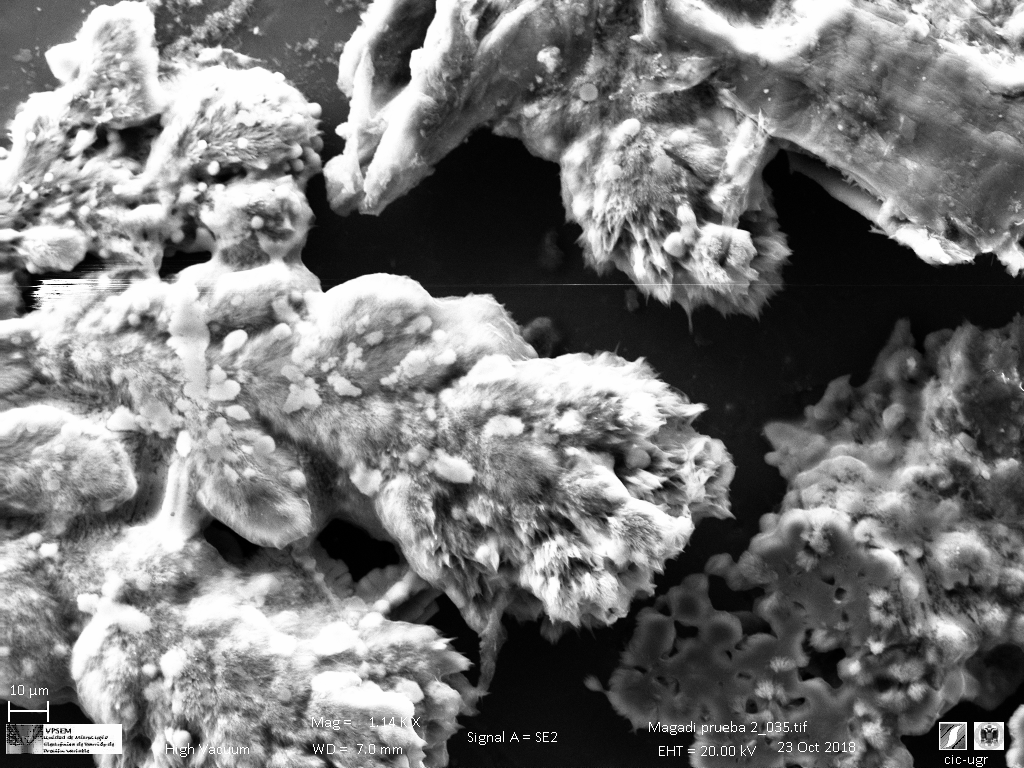

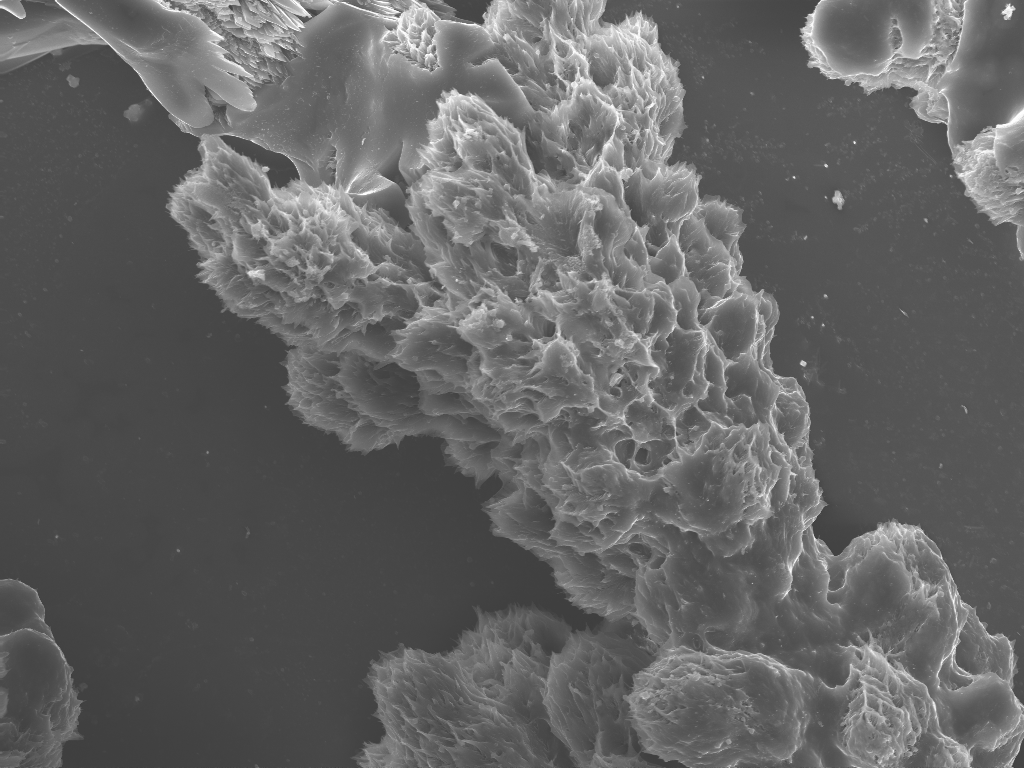


b
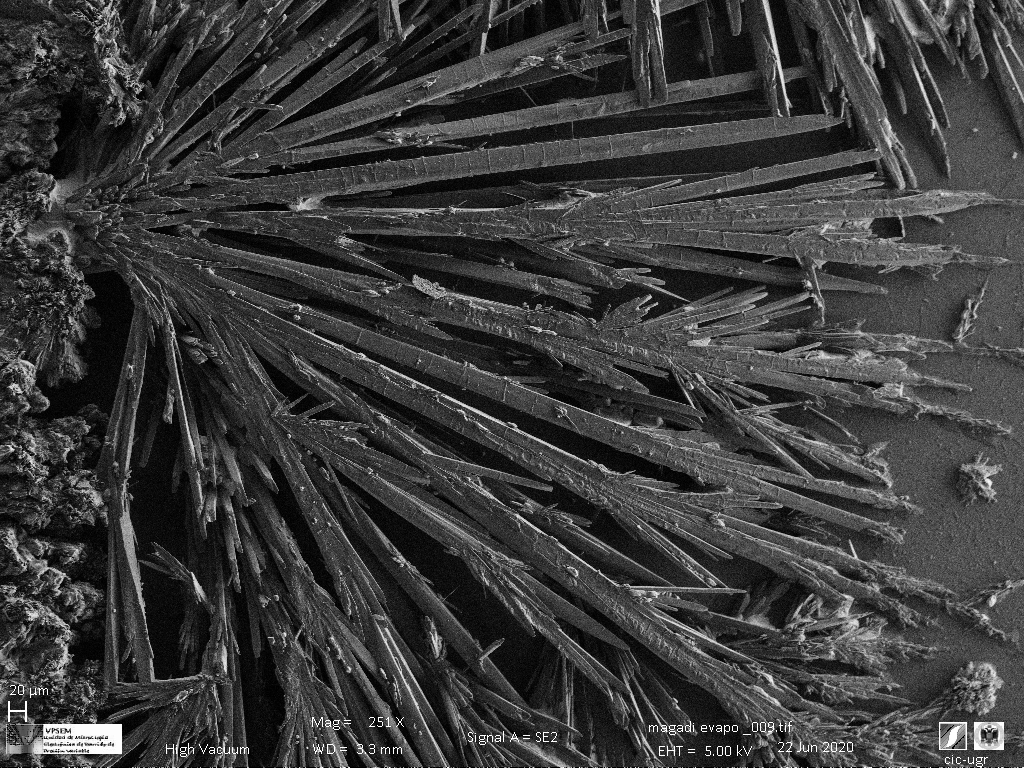

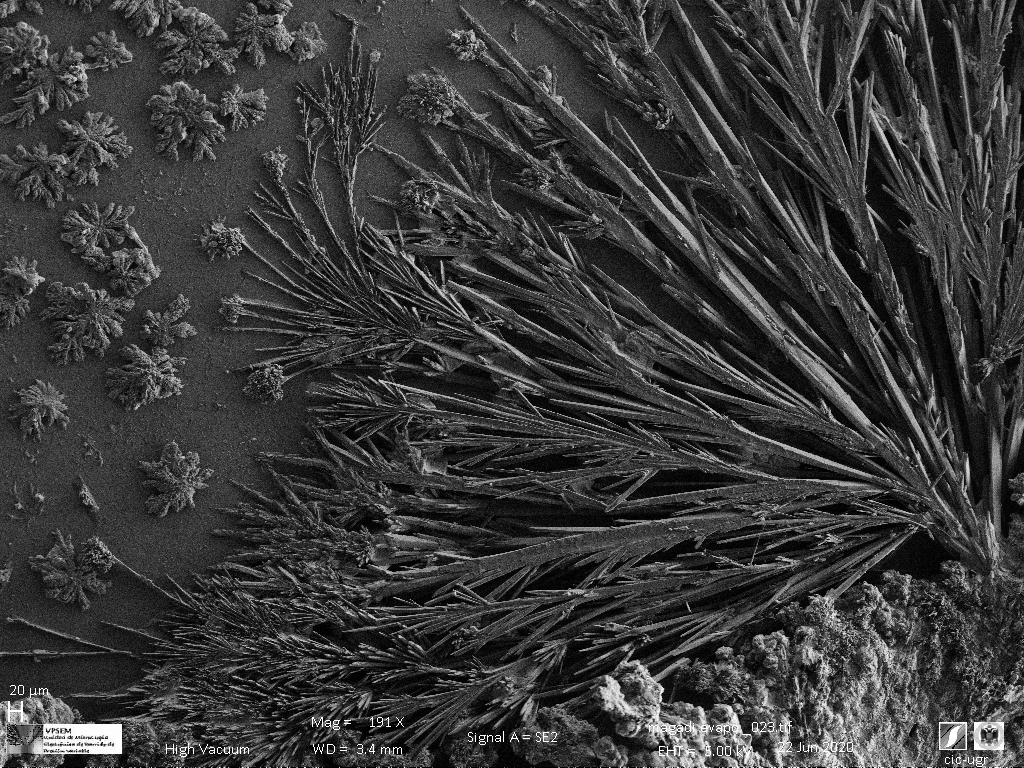

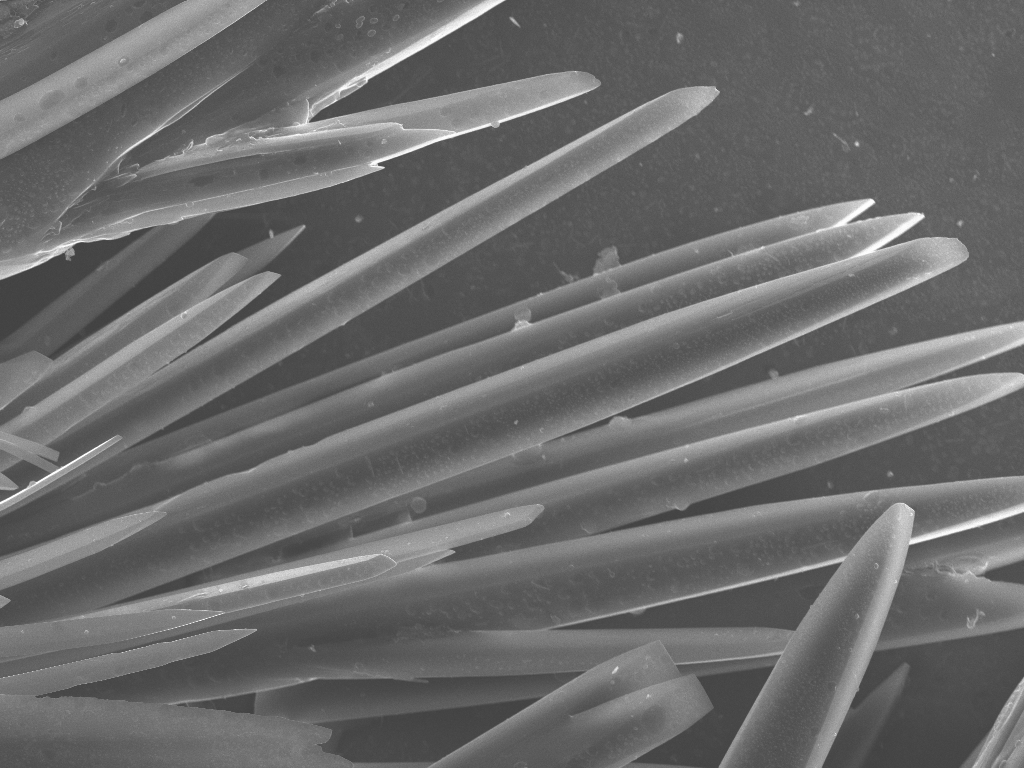


c
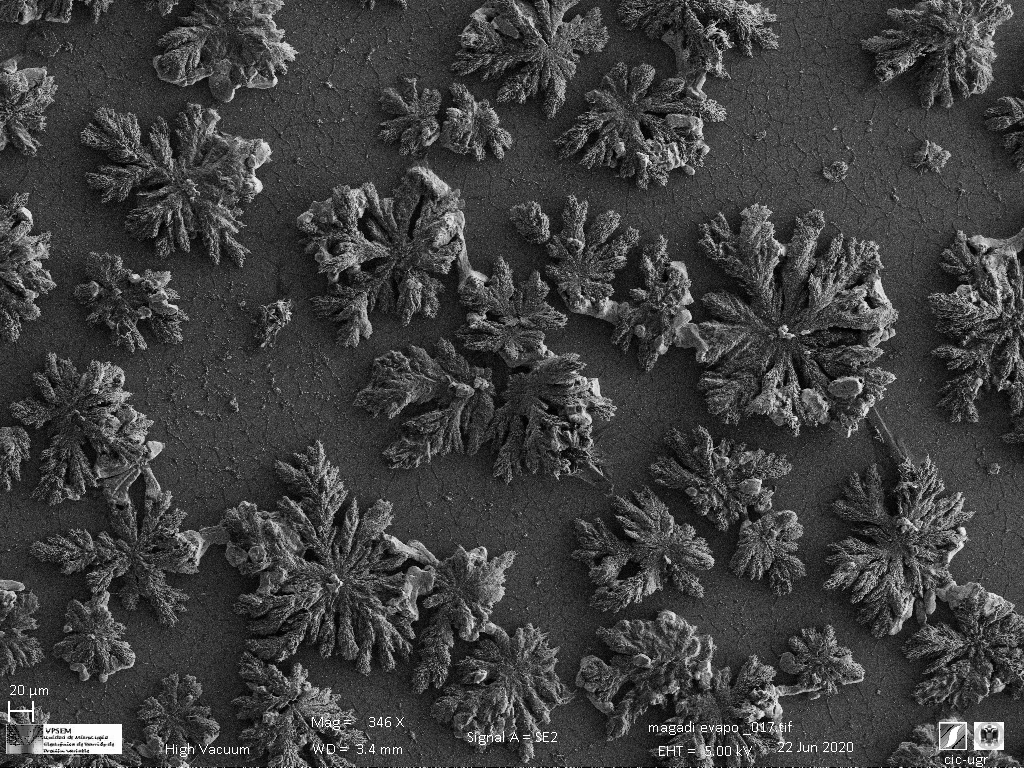

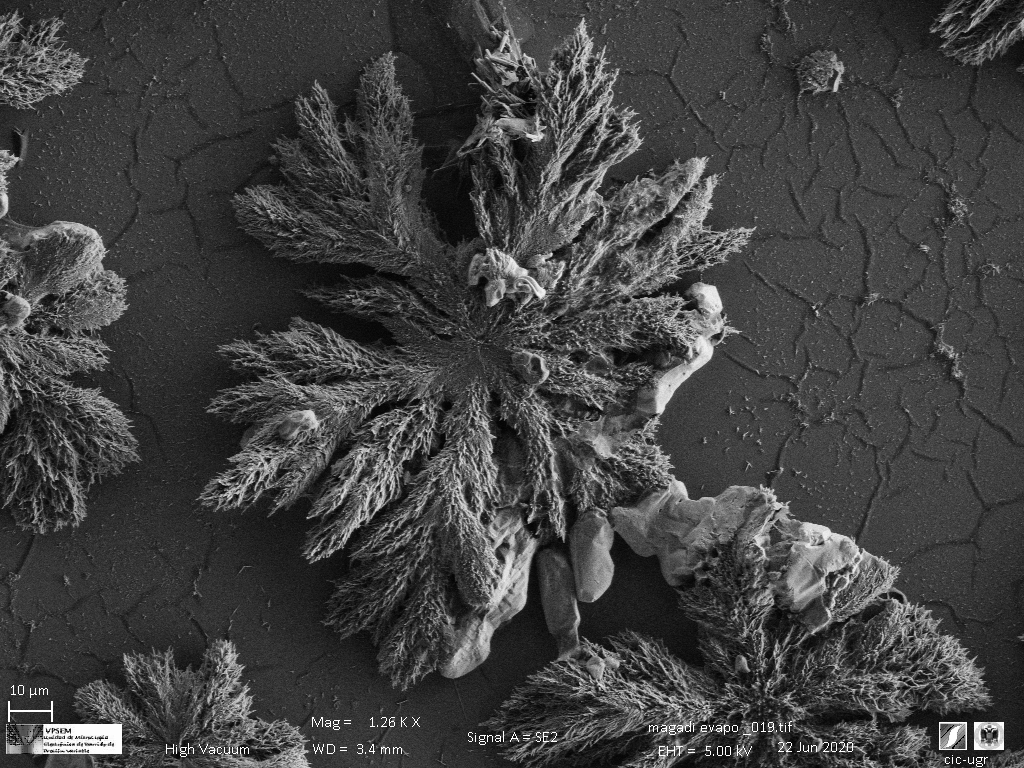

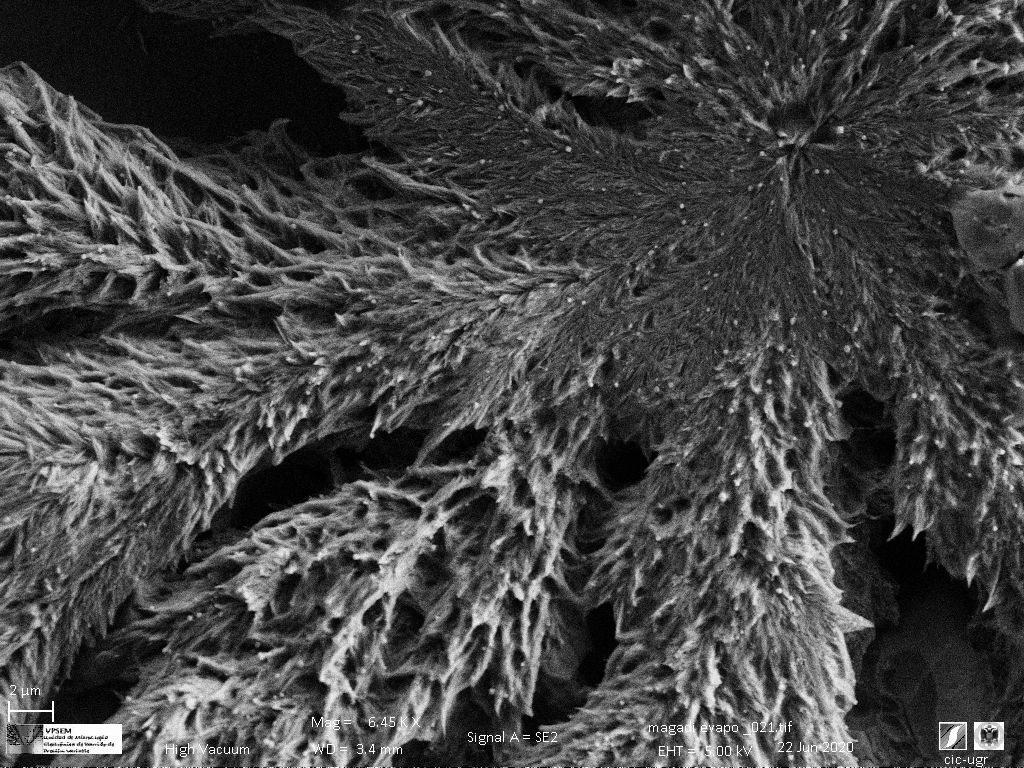


Figure S1: (a) feathery border dendritic minerals, (b) acicular trona, (c) star-like central precipitates with similar feathery texture as shown in panel a


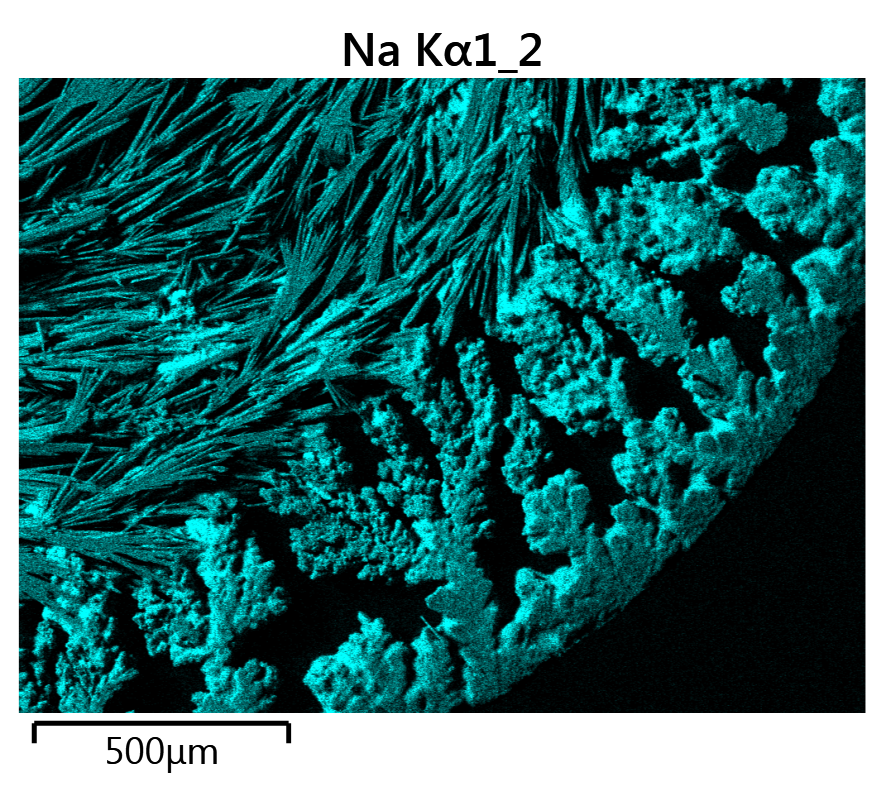

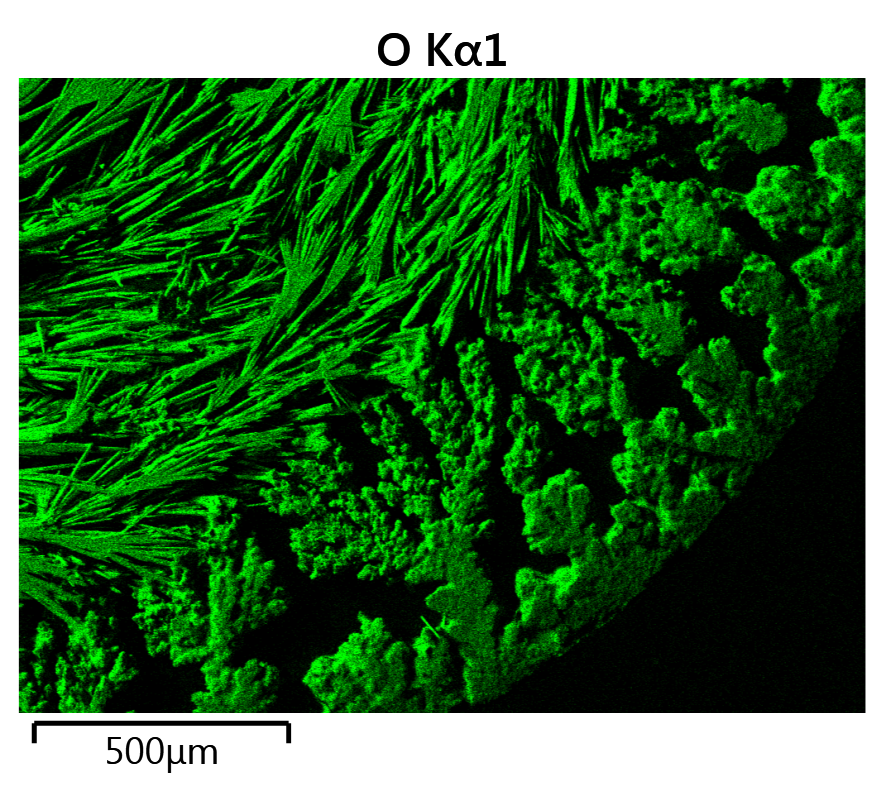

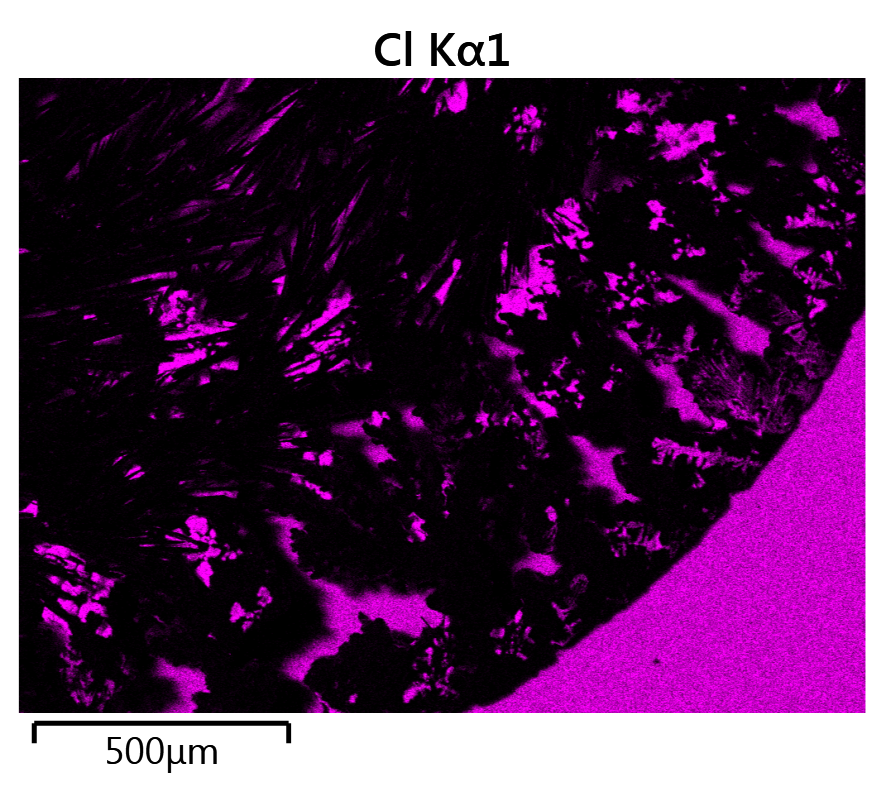


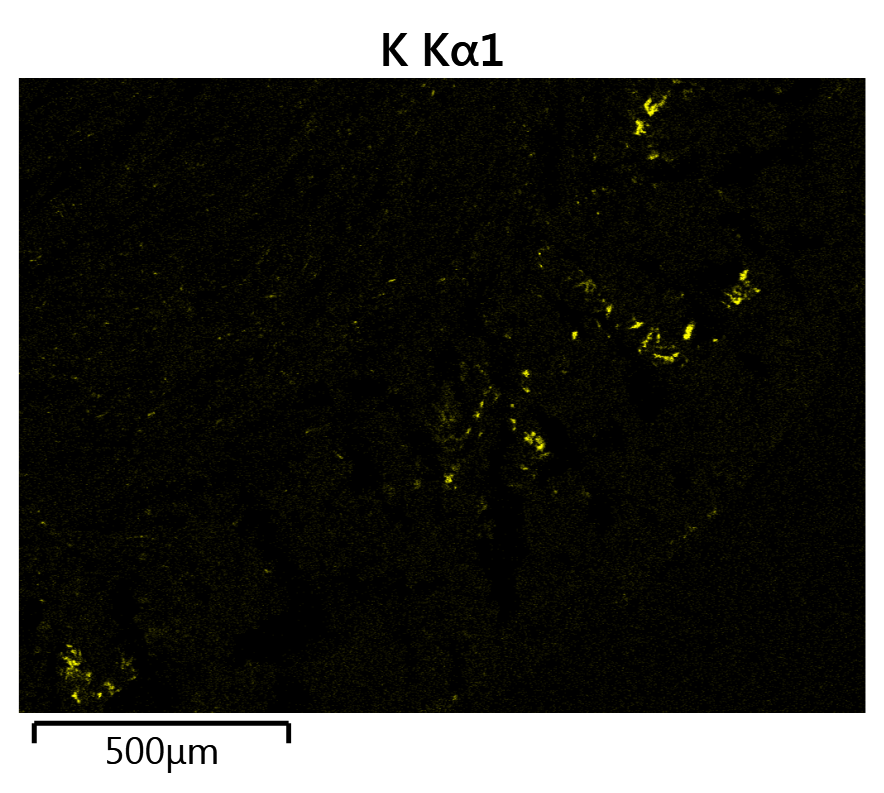

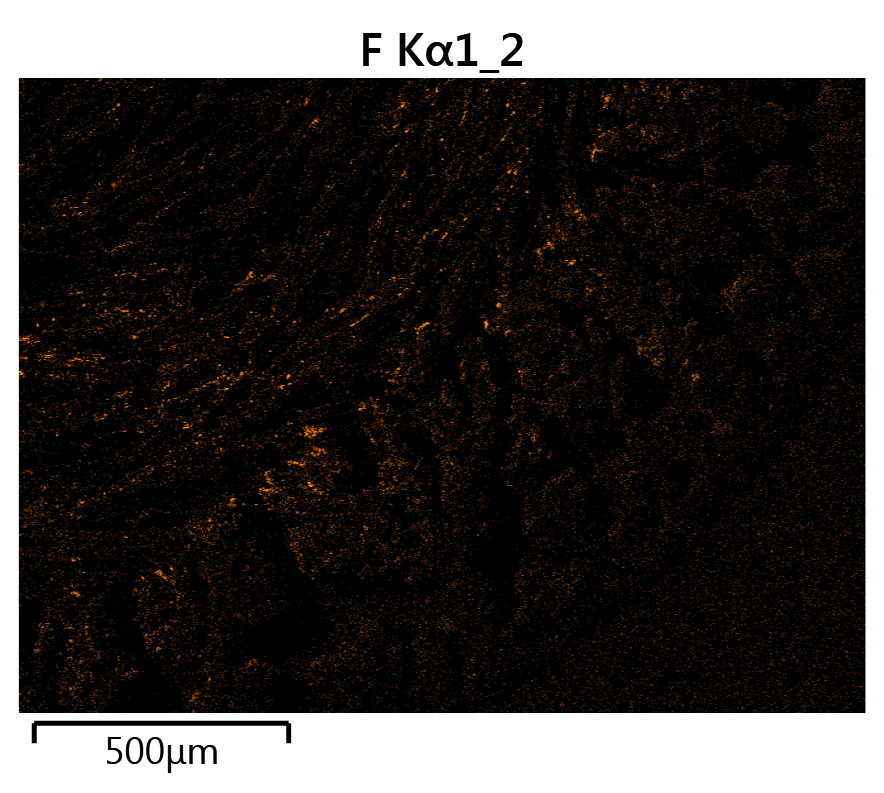


Figure S2: Separate elemental map of the EDX analysis presented in Figure 9a in the main text


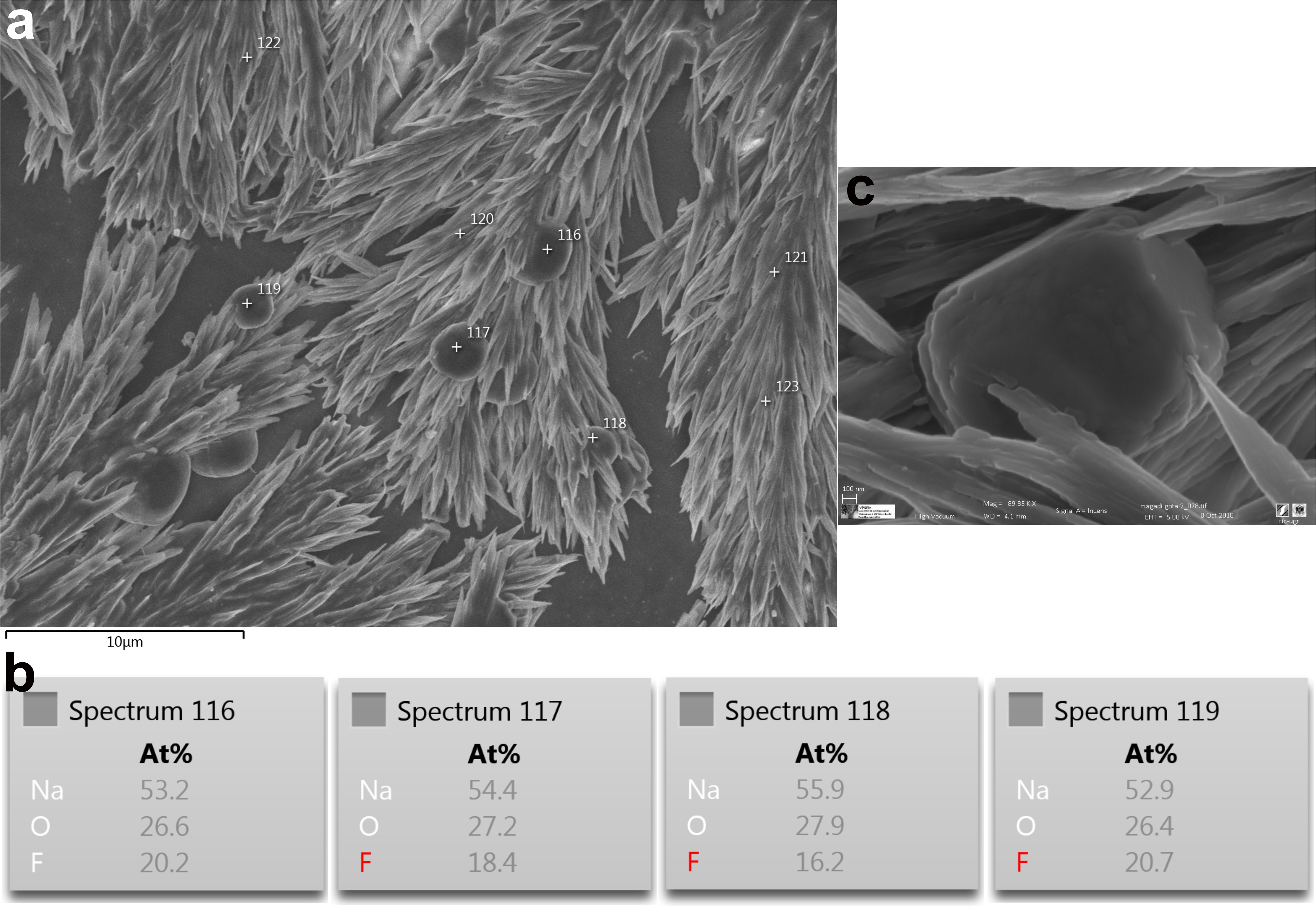


Figure S3: (a) Electron micrograph of rounded and cubic crystals of villiaumite (NaF) between the dendritic trona crystals; (b) EDX elemental analysis of the villiaumite crystals; (c) high-resolution micrograph of semi-rounded cubic villiaumite crystal embedded between trona dendrites

a
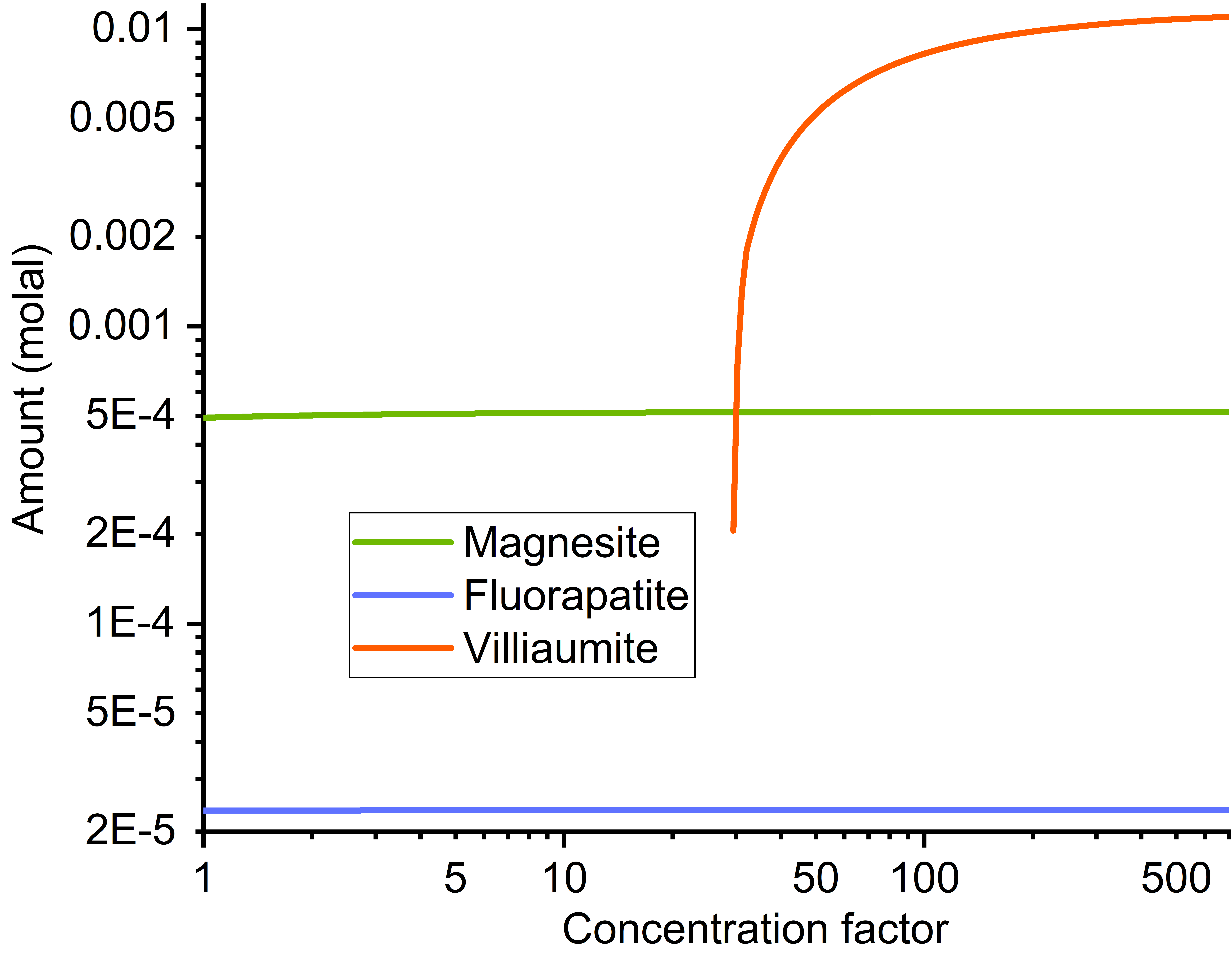
 b
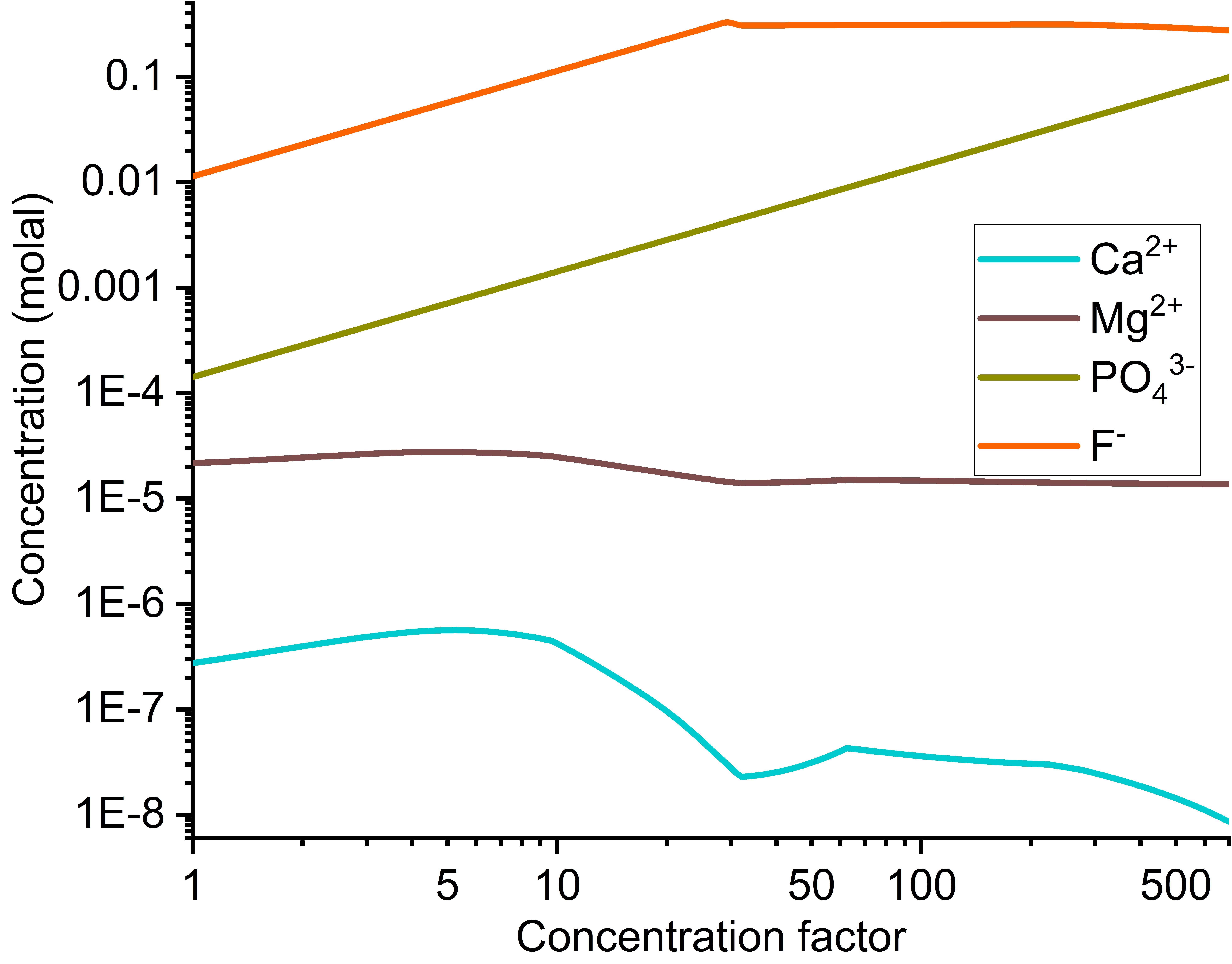


Figure S4: PHREEQC simulation of evaporation and mineral precipitation from Lake Magadi brine at 25 °C in the presence of phosphate and fluoride ions. (a) the mineral precipitation sequence of minor phases and villiaumite and the amount precipitated; (b) chemical evolution of the brine during mineral precipitation




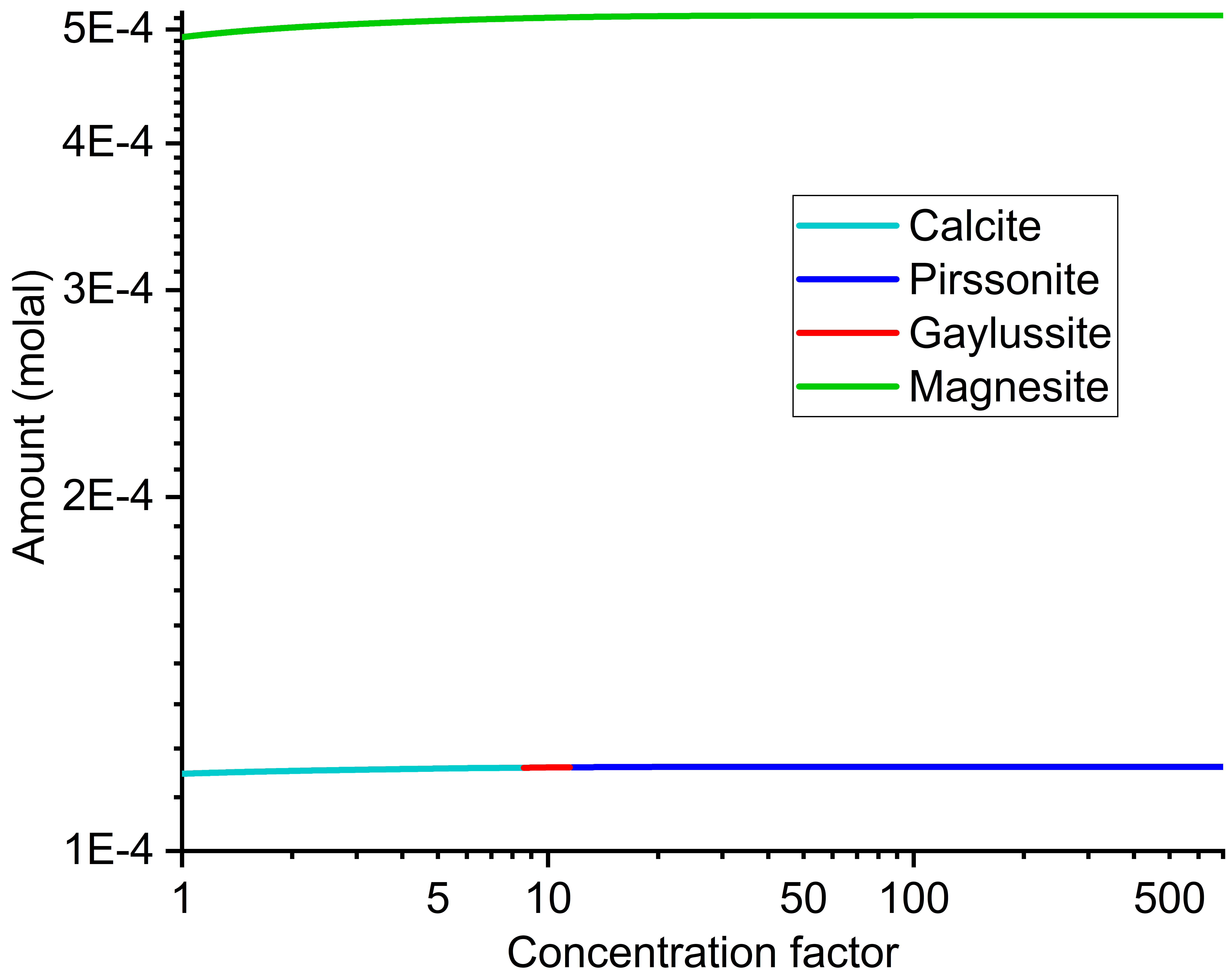




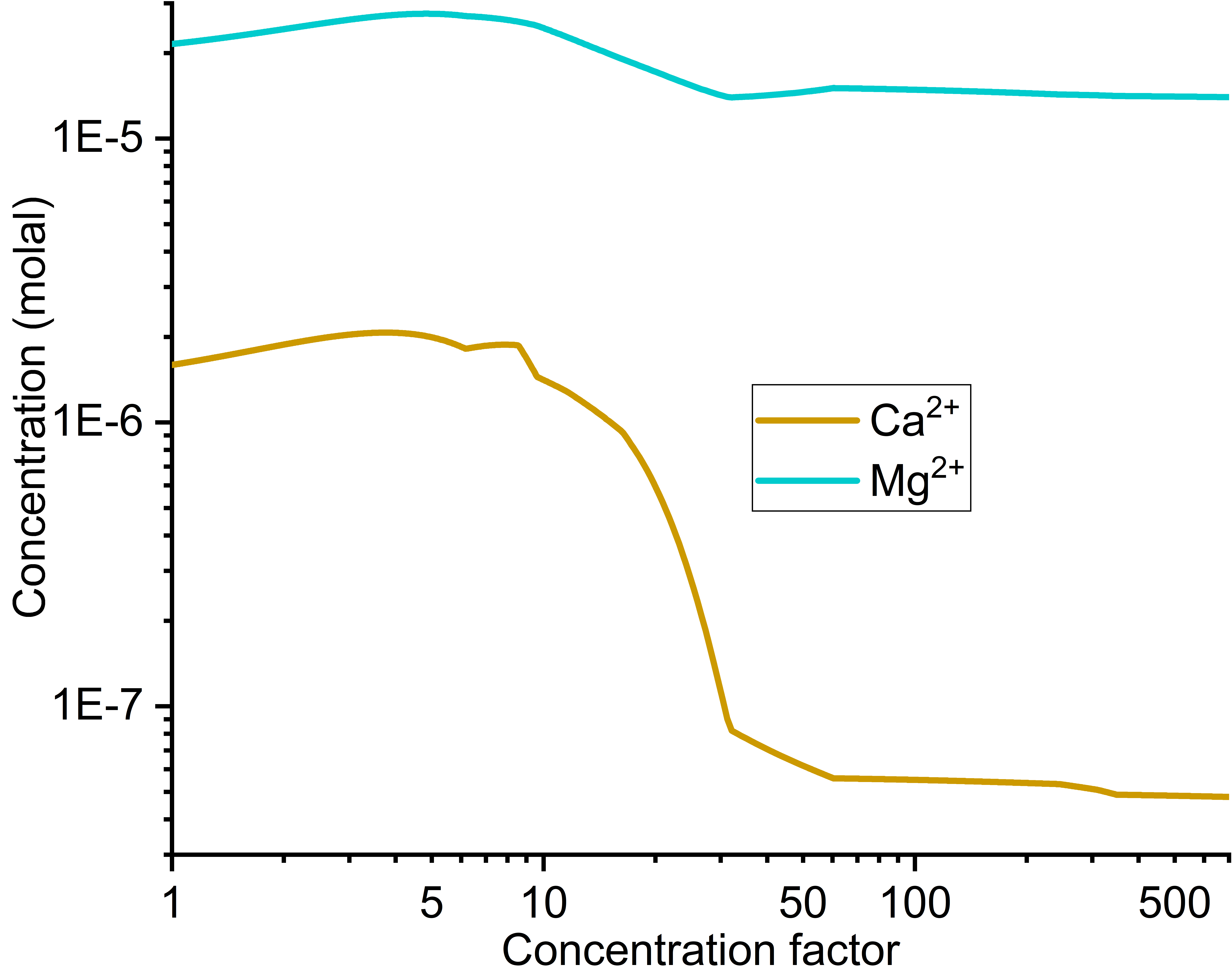


Figure S5: PHREEQC simulation of evaporation and mineral precipitation from Lake Magadi brine at 25 °C in the absence of phosphate and fluoride ions. (a) the mineral precipitation sequence and the precipitated amount of major phases (left) and minor phases (right); (b) chemical evolution of the brine during mineral precipitation

Description of supporting videos

Video S1: Video microscopy of the overall evolution of the precipitation during the evaporation of a single droplet on glass slide. The precipitation process begins on the border of the droplet and progress towards the center (speed 8×, MP4)

Video S2: Detail video microscopy of evaporation and precipitation on the border of a droplet. The precipitation process begins on the border of the droplet and progress towards the center (speed 8×, MP4)

Video S3: Video microscopy of the precipitation process on the border of the droplet marked with red rectangle in Video S2 (speed 4×, MP4)

Video S4: Video microscopy showing the details of the precipitation process at the center of the droplets (speed 2×, MP4)
